# Bioconductor Workflow for Microbiome Data Analysis: from raw reads to community analyses

**DOI:** 10.12688/f1000research.8986.2

**Published:** 2016-11-02

**Authors:** Ben J. Callahan, Kris Sankaran, Julia A. Fukuyama, Paul J. McMurdie, Susan P. Holmes

**Affiliations:** 1 Statistics Department, Stanford University, Stanford, CA, 94305, USA; 2 Whole Biome Inc., San Francisco, CA, 94107, USA

**Keywords:** microbiome, taxonomy, community analysis

## Abstract

High-throughput sequencing of PCR-amplified taxonomic markers (like the 16S rRNA gene) has enabled a new level of analysis of complex bacterial communities known as microbiomes. Many tools exist to quantify and compare abundance levels or OTU composition of communities in different conditions. The sequencing reads have to be denoised and assigned to the closest taxa from a reference database. Common approaches use a notion of 97% similarity and normalize the data by subsampling to equalize library sizes. In this paper, we show that statistical models allow more accurate abundance estimates. By providing a complete workflow in R, we enable the user to do sophisticated downstream statistical analyses, whether parametric or nonparametric. We provide examples of using the R packages dada2, phyloseq, DESeq2, ggplot2 and vegan to filter, visualize and test microbiome data. We also provide examples of supervised analyses using random forests and nonparametric testing using community networks and the ggnetwork package.

## Introduction

The *microbiome* is formed of the ecological communities of microorganisms that dominate the living world. Bacteria can now be identified through the use of next generation sequencing applied at several levels. Shotgun sequencing of all bacteria in a sample delivers knowledge of all the genes present. Here we will only be interested in the identification and quantification of individual taxa (or species) through a ‘fingerprint gene’ called 16s rRNA which is present in all bacteria. This gene presents several variable regions which can be used to identify the different taxa.

Previous standard workflows depended on clustering all 16s rRNA sequences (generated by next generation amplicon sequencing) that occur within a 97% radius of similarity and then assigning these to ‘OTUs’ from reference trees ^2, 3^. These approaches do not incorporate all the data, in particular sequence quality information and statistical information available on the reads were not incorporated into the assignments.

In contrast, the **de novo** read counts used here will be constructed through the incorporation of both the quality scores and sequence frequencies in a probabilistic noise model for nucleotide transitions. For more details on the algorithmic implementation of this step see 4.

After filtering the sequences and removing the chimeræ, the data are compared to a standard database of bacteria and labeled. In this workflow, we have used the labeled sequences to build a de novo phylogenetic with the *phangorn*.

The key step in the sequence analysis is the manner in which reads are denoised and assembled into groups we have chosen to call RSVs (Ribosomal Sequence Variants) instead of the traditional OTUs (Operational Taxonomic Units).

This article describes a computational workflow for performing denoising, filtering, data transformations, visualization, supervised learning analyses, community network tests, hierarchical testing and linear models. We provide all the code and give several examples of different types of analyses and use-cases. There are often many different objectives in experiments involving microbiome data and we will only give a flavor for what could be possible once the data has been imported into R.

In addition, the code can be easily adapted to accommodate batch effects, covariates and multiple experimental factors.

The workflow is based on software packages from the open-source Bioconductor project ^5^. We provide all steps necessary from the denoising and identification of the reads input as raw sequences in fastq files to the comparative testing and multivariate analyses of the samples and analyses of the abundances according to multiple available covariates.

## Methods

### Amplicon bioinformatics: from raw reads to tables

This section demonstrates the “full stack” of amplicon bioinformatics: construction of the sample-by-sequence feature table from the raw reads, assignment of taxonomy, and creation of a phylogenetic tree relating the sample sequences.

First we load the necessary packages.



                        library
                        (
                        "knitr"
                        )

                        library
                        (
                        "BiocStyle"
                        )

                        opts_chunk
                        $
                        set
                        (
                        cache
                         = 
                        FALSE
                        ,
                        fig.path
                        =
                        "dadafigure/"
                        )

                        read_chunk
                        (
                        file.path
                        (
                        "src"
                        , 
                        "bioinformatics.R"
                        ))
                    




                        .cran_packages 
                        <- c
                        (
                        "ggplot2"
                        ,
                         "gridExtra"
                        )
.bioc_packages 
                        <- c
                        (
                        "dada2"
                        , 
                        "phyloseq"
                        , 
                        "DECIPHER"
                        , 
                        "phangorn"
                        )
                    




                        .inst 
                        <- 
                        .cran_packages 
                        %in% 
                        installed.packages
                        ()

                        if
                        (
                        any
                        (
                        !
                        .inst)) {
   
                        install.packages
                        (.cran_packages[
                        !
                        .inst])
}
                    




                        .inst 
                        <- 
                        .bioc_packages 
                        %in% 
                        installed.packages
                        ()

                        if
                        (
                        any
                        (
                        !
                        .inst)) {
   
                        source
                        (
                        "http://bioconductor.org/biocLite.R"
                        )
   
                        biocLite
                        (.bioc_packages[
                        !
                        .inst], 
                        ask
                         = F)
}
                    




                        # Load packages into session, and print package version

                        sapply
                        (
                        c
                        (.cran_packages, .bioc_packages), require, 
                        character.only
                         = 
                        TRUE
                        )
                    




                        set.seed
                        (
                        100
                        )
                    


The data we will analyze here are highly-overlapping Illumina Miseq 2×250 amplicon sequences from the V4 region of the 16S gene ^6^. These 360 fecal samples were collected from 12 mice longitudinally over the first year of life, to investigate the development and stabilization of the murine microbiome ^7^. These data are downloaded from the following location: http://www.mothur.org/MiSeqDevelopmentData/StabilityNoMetaG.tar.



                        miseq_path 
                        <- file.path
                        (
                        "data"
                        , 
                        "MiSeq_SOP"
                        )
filt_path 
                        <- file.path
                        (
                        "data"
                        , 
                        "filtered"
                        )
                    




                        if(!
                        file_test
                        (
                        "-d"
                        , miseq_path)) {
  
                        dir.create
                        (miseq_path)
  
                        download.file
                        (
                        "http://www.mothur.org/MiSeqDevelopmentData/StabilityNoMetaG.tar"
                        ,
                 
                        destfile
                         = 
                        file.path
                        (miseq_path, 
                        "StabilityNoMetaG.tar"
                        ))
  
                        system
                        (
                        paste0
                        (
                        "tar -xvf "
                        , 
                        file.path
                        (miseq_path, 
                        "StabilityNoMetaG.tar"),
                 
                        " -C "
                        , miseq_path, 
                        "/"
                        ))
}
                    




                        fns 
                        <- sort
                        (
                        list.files
                        (miseq_path, 
                        full.names 
                        = 
                        TRUE
                        ))
fnFs 
                        <- 
                        fns[
                        grepl
                        (
                        "R1"
                        , fns)]
fnRs 
                        <- 
                        fns[
                        grepl
                        (
                        "R2"
                        , fns)]
                    


### Trim and Filter

We begin by filtering out low-quality sequencing reads and trimming the reads to a consistent length. While generally recommended filtering and trimming parameters serve as a starting point, no two datasets are identical and therefore it is always worth inspecting the quality of the data before proceeding.

**Figure 1.**
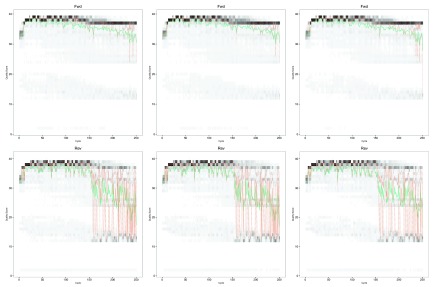
Forward and Reverse Error Profiles, the mean is in green, the median the solid orange line and the quartiles are the dotted orange lines.



                        ii 
                        <- sample
                        (
                        length
                        (fnFs), 
                        3
                        )

                        for
                        (i 
                        in 
                        ii) { 
                        print
                        (
                        plotQualityProfile
                        (fnFs[i]) + 
                        ggtitle
                        (
                        "Fwd"
                        )) }

                        for
                        (i 
                        in 
                        ii) { 
                        print
                        (
                        plotQualityProfile
                        (fnRs[i]) + 
                        ggtitle
                        (
                        "Rev"
                        )) }
                    


Most Illumina sequencing data shows a trend of decreasing average quality towards the end of sequencing reads.

Here, the forward reads maintain high quality throughout, while the quality of the reverse reads drops significantly at about position 160. Therefore, we choose to truncate the forward reads at position 245, and the reverse reads at position 160. We also choose to trim the first 10 nucleotides of each read based on empirical observations across many Illumina datasets that these base positions are particularly likely to contain pathological errors.

We combine these trimming parameters with standard filtering parameters, the most important being the enforcement of a maximum of 2 expected errors per-read ^8^. Trimming and filtering is performed on paired reads jointly, i.e. both reads must pass the filter for the pair to pass.



                        if(!
                        file_test
                        (
                        "-d"
                        , filt_path)) 
                        dir.create
                        (filt_path)

                        filtFs 
                        <- file.path
                        (filt_path, 
                        basename
                        (fnFs))
filtRs 
                        <- file.path
                        (filt_path, 
                        basename
                        (fnRs))

                        for
                        (i 
                        in 
                        seq_along
                        (fnFs)) {
  
                        fastqPairedFilter
                        (
                        c
                        (fnFs[[i]], fnRs[[i]]),
		      
                        c
                        (filtFs[[i]], filtRs[[i]]),
                      
                        trimLeft
                        =
                        10
                        , 
                        truncLen
                        =
                        c
                        (
                        245
                        , 
                        160
                        ),
                      
                        maxN
                        =
                        0
                        , 
                        maxEE
                        =
                        2
                        , 
                        truncQ
                        =
                        2
                        ,
                      
                        compress
                        =
                        TRUE
                        )
}
                    


### Infer sequence variants

After filtering, the typical amplicon bioinformatics workflow clusters sequencing reads into operational taxonomic units (OTUs): groups of sequencing reads that differ by less than a fixed dissimilarity threshhold. Here we instead use the high-resolution DADA2 method to infer ribosomal sequence variants (RSVs) exactly, without imposing any arbitrary threshhold, and thereby resolving variants that differ by as little as one nucleotide ^4^.

The sequence data is imported into R from demultiplexed fastq files (i.e. one fastq for each sample) and simultaneously dereplicated to remove redundancy. We name the resulting derep-class objects by their sample name.

**Figure 2.**
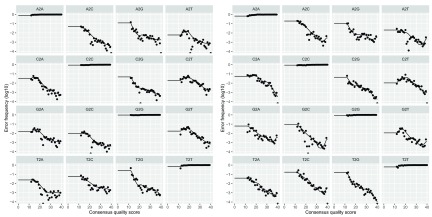
Forward and Reverse Read Error Profiles, showing the frequencies of each type of nucleotide transition as a function of quality.



                        derepFs 
                        <- derepFastq
                        (filtsFs)
derepRs 
                        <- derepFastq
                        (filtsRs)
sam.names 
                        <- sapply
                        (
                        strsplit
                        (
                        basename
                        (filtsFs), 
                        "_"
                        ), `[`, 
                        1
                        )

                        names
                        (derepFs) 
                        <- 
                        sam.names

                        names
                        (derepRs) 
                        <- 
                        sam.names
                    


The DADA2 method relies on a parameterized model of substitution errors to distinguish sequencing errors from real biological variation. Because error rates can (and often do) vary substantially between sequencing runs and PCR protocols, the model parameters can be discovered from the data itself using a form of unsupervised learning in which sample inference is alternated with parameter estimation until both are jointly consistent.

Parameter learning is computationally intensive, as it requires multiple iterations of the sequence inference algorithm, and therefore it is often useful to estimate the error rates from a (sufficiently large) subset of the data.



                        ddF 
                        <- dada
                        (derepFs[
                        1
                        :
                        40
                        ], 
                        err
                        =
                        NULL
                        , 
                        selfConsist
                        =
                        TRUE
                        )


                        ## Initial error matrix unspecified. Error rates will be initialized to the maximum possible estimate
from this data.


                        ## Initializing error rates to maximum possible estimate.
## Sample 1 – 7084 reads in 1955 unique sequences.
## .......
## Sample 40 – 4191 reads in 922 unique sequences.
##    selfConsist step 5
## Convergence after  5 rounds.

ddR 
                        <- dada
                        (derepRs[
                        1
                        :
                        40
                        ], 
                        err
                        =
                        NULL
                        , 
                        selfConsist
                        =
                        TRUE
                        )


                        ## Initial error matrix unspecified. Error rates will be initialized to the maximum possible estimate
from this data.


                        ## Initializing error rates to maximum possible estimate.
## Sample 1 – 7084 reads in 1548 unique sequences.
## .......
## Sample 40 – 4191 reads in 999 unique sequences.
##     selfConsist step 6
## Convergence after 6 rounds.
                    


In order to verify that the error rates have been reasonably well-estimated, we inspect the fit between the observed error rates (black points) and the fitted error rates (black lines) in Figure 2.



                        plotErrors
                        (ddF)

                        plotErrors
                        (ddR)
                    


The DADA2 sequence inference method can run in two different modes: Independent inference by sample (pool=FALSE), and inference from the pooled sequencing reads from all samples (pool=TRUE). Independent inference has the advantage that computation time is linear in the number of samples, and memory requirements are flat with the number of samples. This allows scaling out to datasets of almost unlimited size. Pooled inference is more computationally taxing, and can become intractable for datasets of tens of millions of reads. However, pooling improves the detection of rare variants that were seen just once or twice in an individual sample but many times across all samples. As this dataset is not particularly large, we perform pooled inference. As of version 1.2, multithreading can be activated with the arguments multithread = TRUE, which can substantially speed this step.



                        dadaFs 
                        <- 
                        dada
                        (derepFs, 
                        err
                        =ddF[[
                        1
                        ]]
                        $
                        err_out, 
                        pool
                        =
                        TRUE
                        )
                    




                        ## 362 samples were pooled: 3342527 reads in 272916 unique sequences.
                    




                        dadaRs 
                        <- dada
                        (derepRs, 
                        err
                        =ddR[[
                        1
                        ]]
                        $
                        err_out, 
                        pool
                        =
                        TRUE
                        )
                    




                        ## 362 samples were pooled: 3342527 reads in 278172 unique sequences.
                    


The DADA2 sequence inference step removed (nearly) all substitution and indel errors from the data ^4^. We now merge together the inferred forward and reverse sequences, removing paired sequences that do not perfectly overlap as a final control against residual errors.



                        mergers 
                        <- 
                        mergePairs
                        (dadaFs, derepFs, dadaRs, derepRs)
                    


### Construct sequence table and remove chimeras

The DADA2 method produces a sequence table that is a higher-resolution analogue of the common "OTU table", i.e. a sample by sequence feature table valued by the number of times each sequence was observed in each sample.



                        seqtab.all 
                        <- makeSequenceTable
                        (mergers[
                        !
                        grepl
                        (
                        "Mock"
                        , 
                        names
                        (mergers))])
                    


Notably, chimeras have not yet been removed. The error model in the sequence inference algorithm does not include a chimera component, and therefore we expect this sequence table to include many chimeric sequences. We now remove chimeric sequences by comparing each inferred sequence to the others in the table, and removing those that can be reproduced by stitching together two more abundant sequences.



                        seqtab 
                        <- removeBimeraDenovo
                        (seqtab.all)
                    


Although exact numbers vary substantially by experimental condition, it is typical that chimeras comprise a substantial fraction of inferred sequence variants, but only a small fraction of all reads. That is what is observed here: 1503 of 1892 sequence variants were chimeric, but these only represented 10% of all reads.

### Assign taxonomy

One of the benefits of using well-classified marker loci like the 16S rRNA gene is the ability to taxonomically classify the sequence variants. The dada2 package implements the naive Bayesian classifier method for this purpose ^9^. This classifier compares sequence variants to a training set of classified sequences, and here we use the RDP v14 training set ^10^.



                        ref_fasta 
                        <- 
                        "data/rdp_train_set_14.fa.gz"

                        taxtab 
                        <- assignTaxonomy
                        (seqtab, 
                        refFasta 
                        = ref_fasta)

                        colnames
                        (taxtab) 
                        <- c
                        (
                        "Kingdom"
                        , 
                        "Phylum"
                        , 
                        "Class"
                        , 
                        "Order"
                        , 
                        "Family"
                        , 
                        "Genus"
                        )
                    


GreenGenes and Silva training set fasta files formatted for the assignTaxonomy function are also available for download at https://www.dropbox.com/sh/mfcivbudmc21cqt/AAB1l-AUM5uKvjrR33ct-cTXa?dl=0.

### Construct phylogenetic tree

Phylogenetic relatedness is commonly used to inform downstream analyses, especially the calculation of phylogeny-aware distances between microbial communities. The DADA2 sequence inference method is reference-free, so we must construct the phylogenetic tree relating the inferred sequence variants de novo. We begin by performing a multiple-alignment using the DECIPHER R package ^11^.



                        seqs 
                        <- getSequences
                        (seqtab)

                        names
                        (seqs) 
                        <- 
                        seqs 
                        # This propagates to the tip labels of the tree

                        alignment 
                        <- AlignSeqs
                        (
                        DNAStringSet
                        (seqs)
                        , 
                        anchor=
                        NA
                        )
                    




                        ## Determining distance matrix based on shared 5-mers:
##
## Clustering into groups by similarity:
##
## Aligning Sequences:
##
## Determining distance matrix based on alignment:
##
## Reclustering into groups by similarity:
##
## Realigning Sequences:
##
## Refining the alignment:
                    


The *phangorn* R package is then used to construct a phylogenetic tree. Here we first construct a neighbor-joining tree, and then fit a GTR+G+I (Generalized time-reversible with Gamma rate variation) maximum likelihood tree using the neighbor-joining tree as a starting point.



                        phang.align 
                        <- phyDat
                        (
                        as
                        (alignment, 
                        "matrix"
                        ), 
                        type
                        =
                        "DNA"
                        )

                        dm 
                        <- dist.ml
                        (phang.align)
treeNJ 
                        <- NJ
                        (dm) 
                        # Note, tip order != sequence order

                        fit 
                        = pml
                        (treeNJ, 
                        data
                        =phang.align)


                        ## negative edges length changed to 0!


                        fitGTR 
                        <- update
                        (fit, 
                        k
                        =
                        4
                        , 
                        inv
                        =
                        0.2
                        )
fitGTR 
                        <- optim.pml
                        (fitGTR, 
                        model
                        =
                        "GTR"
                        , 
                        optInv
                        =
                        TRUE
                        , 
                        optGamma
                        =
                        TRUE
                        ,
                      
                        rearrangement 
                        = 
                        "stochastic"
                        , 
                        control 
                        = 
                        pml.control
                        (
                        trace 
                        = 
                        0
                        ))

                        detach
                        (
                        "package:phangorn"
                        , 
                        unload
                        =
                        TRUE
                        )
                    


### Combine data into a phyloseq object

The *phyloseq* package organizes and synthesizes the different data types from a typical amplicon sequencing experiment into a single data object that can be easily manipulated. The last bit of information needed is the sample data contained in a .csv file.



                        mimarks_path 
                        <- 
                        "data/MIMARKS_Data_combined.csv"

                        samdf 
                        <- read.csv
                        (mimarks_path, 
                        header
                        =
                        TRUE
                        )
samdf
                        $
                        SampleID 
                        <- paste0
                        (
                        gsub
                        (
                        "00"
                        , 
                        ""
                        , samdf
                        $
                        host_subject_id), 
                        "D"
                        , samdf
                        $
                        age-
                        21
                        )
samdf 
                        <- 
                        samdf[
                        !
                        duplicated
                        (samdf
                        $
                        SampleID),] 
                        # Remove dupicate entries for reverse reads

                        rownames
                        (seqtab) 
                        <- gsub
                        (
                        "124"
                        , 
                        "125"
                        , 
                        rownames
                        (seqtab)) 
                        # Fixing an odd discrepancy

                        all
                        (
                        rownames
                        (seqtab) 
                        %in% 
                        samdf
                        $
                        SampleID) 
                        # TRUE


                        ## [1] TRUE


                        rownames
                        (samdf) 
                        <- 
                        samdf
                        $
                        SampleID
keep.cols 
                        <- c
                        (
                        "collection_date"
                        , 
                        "biome"
                        , 
                        "target_gene"
                        , 
                        "target_subfragment"
                        ,

                        "host_common_name"
                        , 
                        "host_subject_id"
                        , 
                        "age"
                        , 
                        "sex"
                        , 
                        "body_product"
                        , 
                        "tot_mass"
                        ,

                        "diet"
                        , 
                        "family_relationship"
                        , 
                        "genotype"
                        , 
                        "SampleID"
                        ) 

                        samdf 
                        <- 
                        samdf[
                        rownames
                        (seqtab), keep.cols]
                    


The full suite of data for this study – the sample-by-sequence feature table, the sample metadata, the sequence taxonomies, and the phylogenetic tree – can now be combined into a single object.



                        ps 
                        <- phyloseq
                        (
                        tax_table
                        (taxtab), 
                        sample_data
                        (samdf),
                 
                        otu_table
                        (seqtab, 
                        taxa_are_rows 
                        = 
                        FALSE
                        ),
                        phy_tree
                        (fitGTR
                        $
                        tree))
                    


### phyloseq

*phyloseq* ^12^ is an R package to import, store, analyze, and graphically display complex phylogenetic sequencing data that has already been clustered into Operational Taxonomic Units (OTUs) or more appropriately denoised, and it is most useful when there is also associated sample data, phylogeny, and/or taxonomic assignment of each taxa. *phyloseq* leverages and builds upon many of the tools available in R for ecology and phylogenetic analysis ( *vegan* ^13^, *ade4* ^14^, *ape* ^15^), while also using advanced/flexible graphic systems ( *ggplot2* ^16^) to easily produce publication-quality graphics of complex phylogenetic data. The phyloseq package uses a specialized system of S4 data classes to store all related phylogenetic sequencing data as a single, self-consistent, self-describing experiment-level object, making it easier to share data and reproduce analyses. In general, phyloseq seeks to facilitate the use of R for efficient interactive and reproducible analysis of amplicon count data jointly with important sample covariates.

### Further documentation

This tutorial shows a useful example workflow, but many more analyses are available to you in phyloseq, and R in general, than can fit in a single workflow. The phyloseq home page is a good place to begin browsing additional phyloseq documentation, as are the three vignettes included within the package, and linked directly at the phyloseq release page on Bioconductor.

### Loading data

Many use cases result in the need to import and combine different data into a phyloseq class object, this can be done using the import_biom function to read recent QIIME format files, older files can still be imported with import_qiime. More complete details can be found on the phyloseq FAQ page.

In the previous section the results of *dada2* sequence processing were organized into a phyloseq object. This object was also saved in R-native serialized RDS format. We will re-load this here for completeness as the initial object p0.



                        library
                        (
                        "phyloseq"
                        )

                        library
                        (
                        "gridExtra"
                        )
ps 
                        = readRDS(
                        "data/ps.rds"
                        )
ps

## phyloseq-class experiment-level object
## otu_table()   OTU Table:         [ 389 taxa and 360 samples ]
## sample_data() Sample Data:       [ 360 samples by 14 sample variables ]
## tax_table()   Taxonomy Table:    [ 389 taxa by 6 taxonomic ranks ]
## phy_tree()    Phylogenetic Tree: [ 389 tips and 387 internal nodes ]
                    


### Shiny-phyloseq

It can be beneficial to start the data exploration process interactively, this often saves time in detecting outliers and specific features of the data. Shiny-phyloseq ^17^ is an interactive web application that provides a graphical user interface to the phyloseq package. The object just loaded into the R session in this workflow is suitable for this graphical interaction with Shiny-phyloseq.

### Filtering

*phyloseq* provides useful tools for filtering, subsetting, and agglomerating taxa – a task that is often appropriate or even necessary for effective analysis of microbiome count data. In this subsection, we graphically explore the prevalence of taxa in the example dataset, and demonstrate how this can be used as a filtering criteria. One of the reasons to filter in this way is to avoid spending much time analyzing taxa that were seen only rarely among samples. This also turns out to be a useful filter of noise (taxa that are actually just artifacts of the data collection process), a step that should probably be considered essential for datasets constructed via heuristic OTU-clustering methods, which are notoriously prone to generating spurious taxa.

### Taxonomic filtering

In many biological settings, the set of all organisms from all samples are well-represented in the available taxonomic reference database. When (and only when) this is the case, it is reasonable or even advisable to filter taxonomic features for which a high-rank taxonomy could not be assigned. Such ambiguous features in this setting are almost always sequence artifacts that don’t exist in nature. It should be obvious that such a filter is not appropriate for samples from poorly characterized or novel specimens, at least until the possibility of taxonomic novelty can be satisfactorily rejected. Phylum is a useful taxonomic rank to consider using for this purpose, but others may work effectively for your data.

To begin, create a table of read counts for each Phylum present in the dataset.



                        # Show available ranks in the dataset

                        rank_names
                        (ps)
                    




                        ## [1] "Kingdom" "Phylum" "Class" "Order" "Family" "Genus"
                    




                        # Create table, number of features for each phyla

                        table
                        (
                        tax_table
                        (ps)[
                        , 
                        "Phylum"
                        ], 
                        exclude 
                        = 
                        NULL
                        )
                    




                        ##

                        ##              Actinobacteria              Bacteroidetes

                        ##                          13                         23

                        ## Candidatus_Saccharibacteria  Cyanobacteria/Chloroplast

                        ##                           1                          4

                        ##         Deinococcus-Thermus                 Firmicutes

                        ##                           1                        327

                        ##                Fusobacteria             Proteobacteria

                        ##                           1                         11

                        ##                 Tenericutes            Verrucomicrobia

                        ##                           1                          1

                        ##                        <NA>

                        ##                           6
                    


This shows a few phyla for which only one feature was observed. Those may be worth filtering, and we’ll check that next. First, notice that in this case, six features were annotated with a Phylum of NA. These features are probably artifacts in a dataset like this, and should be removed.

The following ensures that features with ambiguous phylum annotation are also removed. Note the flexibility in defining strings that should be considered ambiguous annotation.



                        ps0 
                        <- 
                        subset_taxa
                        (ps, 
                        !
                        is.na
                        (Phylum) 
                        & !
                        Phylum 
                        %in% 
                        c
                        (
                        ""
                        , 
                        "uncharacterized"
                        ))
                    


A useful next step is to explore feature *prevalence* in the dataset, which we will define here as the number of samples in which a taxa appears at least once.



                        # Compute prevalence of each feature, store as data.frame

                        prevdf 
                        = 
                        apply
                        (
                        X 
                        = 
                        otu_table
                        (ps0),
                 
                        MARGIN 
                        = 
                        ifelse
                        (
                        taxa_are_rows
                        (ps0), 
                        yes 
                        = 
                        1
                        , 
                        no 
                        = 
                        2
                        ),
                 
                        FUN = 
                        function
                        (
                        x
                        ){
                        sum
                        (x > 
                        0
                        )})

                        # Add taxonomy and total read counts to this data.frame

                        prevdf 
                        = data.frame
                        (
                        Prevalence 
                        = prevdf,
                      
                        TotalAbundance 
                        = 
                        taxa_sums
                        (ps0),
                      
                        tax_table
                        (ps0))
                    


Are there phyla that are comprised of mostly low-prevalence features? Compute the total and average prevalences of the features in each phylum.



                        plyr::
                        ddply
                        (prevdf, 
                        "Phylum"
                        , 
                        function
                        (
                        df1
                        ){
                        cbind
                        (
                        mean
                        (df1$
                        Prevalence),
                        sum
                        (df1$
                        Prevalence))})
                    




                        ##                          Phylum       1      2
## 1                Actinobacteria   120.2   1562
## 2                 Bacteroidetes   265.5   6107
## 3   Candidatus_Saccharibacteria   280.0    280
## 4     Cyanobacteria/Chloroplast    64.2    257
## 5           Deinococcus-Thermus    52.0     52
## 6                    Firmicutes   179.2  58614
## 7                  Fusobacteria     2.0      2
## 8                Proteobacteria    59.1    650
## 9                   Tenericutes   234.0    234
## 10              Verrucomicrobia   104.0    104
                    


Deinococcus-Thermus appeared in just over one percent of samples, and Fusobacteria appeared in just 2 samples total. In some cases it might be worthwhile to explore these two phyla in more detail despite this (though probably not Fusobacteria’s two samples). For the purposes of this example, though, they will be filtered from the dataset.



                        # Define phyla to filter

                        filterPhyla 
                        = c
                        (
                        "Fusobacteria"
                        , 
                        "Deinococcus-Thermus"
                        )

                        # Filter entries with unidentified Phylum.

                        ps1 
                        = subset_taxa
                        (ps0, !
                        Phylum %in% 
                        filterPhyla)
ps1

## phyloseq-class experiment-level object
## otu_table()   OTU Table:         [ 381 taxa and 360 samples ]
## sample_data() Sample Data:       [ 360 samples by 14 sample variables ]
## tax_table()   Taxonomy Table:    [ 381 taxa by 6 taxonomic ranks ]
## phy_tree()    Phylogenetic Tree: [ 381 tips and 379 internal nodes ]
                    


### Prevalence Filtering

The previous filtering steps are considered *supervised*, because they relied on prior information that is external to this experiment (a taxonomic reference database). This next filtering step is completely *unsupervised*, relying only on the data in this experiment, and a parameter that we will choose after exploring the data. Thus, this filtering step can be applied even in settings where taxonomic annotation is unavailable or unreliable.

First, explore the relationship of prevalence and total read count for each feature. Sometimes this reveals outliers that should probably be removed, and also provides insight into the ranges of either feature that might be useful. This aspect depends quite a lot on the experimental design and goals of the downstream inference, so keep these in mind. It may even be the case that different types of downstream inference require different choices here. There is no reason to expect ahead of time that a single filtering workflow is appropriate for all analysis.



                        # Subset to the remaining phyla

                        prevdf1 
                        = 
                        subset
                        (prevdf, Phylum %in% 
                        get_taxa_unique
                        (ps1, 
                        "Phylum"
                        ))

                        ggplot
                        (prevdf1, 
                        aes
                        (TotalAbundance, Prevalence / 
                        nsamples
                        (ps0),
                        color
                        =Phylum)) +
  
                        # Include a guess for parameter
  
                        geom_hline
                        (
                        yintercept 
                        = 
                        0.05
                        , 
                        alpha 
                        = 
                        0.5
                        , 
                        linetype 
                        = 
                        2
                        ) + 
                        geom_point
                        (
                        size 
                        = 
                        2
                        , 
                        alpha 
                        = 
                        0.7
                        ) +
  
                        scale_x_log10
                        () +  
                        xlab
                        (
                        "Total Abundance"
                        ) + 
                        ylab
                        (
                        "Prevalence [Frac. Samples]"
                        ) +
  
                        facet_wrap
                        (~Phylum) + 
                        theme
                        (
                        legend.position
                        =
                        "none"
                        )
                    


Sometimes a natural separation in the dataset reveals itself, or at least, a conservative choice that is in a stable region for which small changes to the choice would have minor or no effect on the biological interpreation (stability). Here no natural separation is immediately evident, but it looks like we might reasonably define a prevalence threshold in a range of zero to 10 percent or so. Take care that this choice does not introduce bias into a downstream analysis of association of differential abundance.

The following uses five percent of all samples as the prevalence threshold.

**Figure 3.**
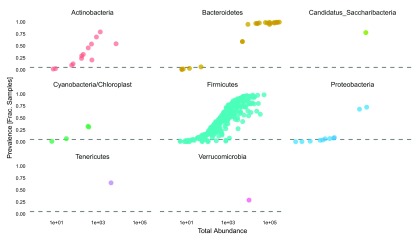
Taxa prevalence versus total counts. Each point is a different taxa. Exploration of the data in this way is often useful for selecting filtering parameters, like the minimum prevalence criteria we will used to filter the data above.



                        #  Define prevalence threshold as 5% of total samples

                        prevalenceThreshold 
                        = 
                        0.05 
                        * 
                        nsamples
                        (ps0)
prevalenceThreshold

## [1] 18


                        # Execute prevalence filter, using `prune_taxa()` function

                        keepTaxa 
                        = rownames
                        (prevdf1)[(prevdf1
                        $
                        Prevalence >= 
                        prevalenceThreshold)]

                        ps2 
                        = prune_taxa
                        (keepTaxa, ps0)
                    


### Agglomerate taxa

When there is known to be a lot of species or sub-species functional redundancy in a microbial community, it might be useful to agglomerate the data features corresponding to closely related taxa. Ideally we would know the functional redundancies perfectly ahead of time, in which case we would agglomerate taxa using those defined relationships and the merge_taxa() function in phyloseq. That kind of exquisite functional data is usually not available, and different pairs of microbes will have different sets of overlapping functions, complicating the matter of defining appropriate grouping criteria.

While not necessarily the most useful or functionally-accurate criteria for grouping microbial features (sometimes far from accurate), taxonomic agglomeration has the advantage of being much easier to define ahead of time. This is because taxonomies are usually defined with a comparatively simple tree-like graph structure that has a fixed number of internal nodes, called “ranks”. This structure is simple enough for the phyloseq package to represent taxonomies as table of taxonomy labels. Taxonomic agglomeration groups all the “leaves” in the hierarchy that descend from the user-prescribed agglomerating rank, this is sometimes called ‘glomming’.

The following example code shows how one would combine all features that descend from the same genus.



                        # How many genera would be present after filtering?

                        length
                        (
                        get_taxa_unique
                        (ps2, 
                        taxonomic.rank 
                        = 
                        "Genus"
                        ))

## [1] 49

ps3 
                        = tax_glom
                        (ps2, 
                        "Genus"
                        , 
                        NArm 
                        = 
                        TRUE
                        )
                    


If taxonomy is not available or not reliable, tree-based agglomeration is a "taxonomy-free" alternative to combine data features corresponding to closely-related taxa. In this case, rather than taxonomic rank, the user specifies a tree height corresponding to the phylogenetic distance between features that should define their grouping. This is very similar to “OTU Clustering”, except that in many OTU Clustering algorithms the sequence distance being used does not have the same (or any) evolutionary definition.



                        h1 
                        = 
                        0.4

                        ps4 
                        = tip_glom
                        (ps2, 
                        h 
                        = h1)
                    


Here phyloseq’s plot_tree() function compare the original unfiltered data, the tree after taxonoic agglomeration, and the tree after phylogenetic agglomeration. These are stored as separate plot objects, then rendered together in one combined graphic using gridExtra::grid.arrange.



                        multiPlotTitleTextSize 
                        = 
                        8

                        p2tree 
                        = plot_tree
                        (ps2, 
                        method 
                        = 
                        "treeonly"
                        ,
                     
                        ladderize 
                        = 
                        "left"
                        ,
                     
                        title 
                        = 
                        "Before Agglomeration"
                        ) 
                        +
  
                        theme
                        (
                        plot.title 
                        = 
                        element_text
                        (
                        size 
                        = multiPlotTitleTextSize))
p3tree 
                        = plot_tree
                        (ps3, 
                        method 
                        = 
                        "treeonly"
                        ,
                     
                        ladderize 
                        = 
                        "left"
                        , 
                        title 
                        = 
                        "By Genus"
                        ) 
                        +
  
                        theme
                        (
                        plot.title 
                        = 
                        element_text
                        (
                        size 
                        = multiPlotTitleTextSize))
p4tree 
                        = plot_tree
                        (ps4, 
                        method 
                        = 
                        "treeonly"
                        ,
                     
                        ladderize 
                        = 
                        "left"
                        , 
                        title 
                        = 
                        "By Height"
                        ) 
                        +
  
                        theme
                        (
                        plot.title 
                        = 
                        element_text
                        (
                        size 
                        = multiPlotTitleTextSize))


                        # group plots together

                        grid.arrange
                        (
                        nrow 
                        = 
                        1
                        , p2tree, p3tree, p4tree)
                    


**Figure 4.**
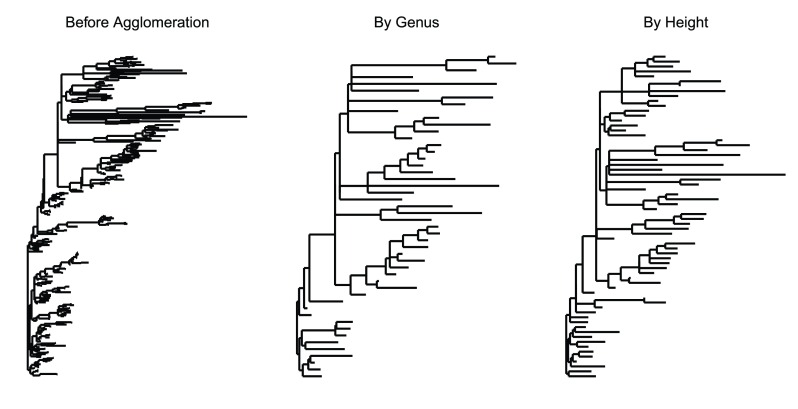
The original tree (left), taxonomic agglomeration at Genus rank (middle), phylogentic agglomeration at a fixed distance of 0.4 (right).

### Abundance value transformation

It is usually necessary to transform microbiome count data to account for differences in library size, variance, scale, etc. The phyloseq package provides a flexible interface for defining new functions to accomplish these transformations of the abundance values via the transform_sample_counts() function. The first argument to this function is the phyloseq object you want to transform, and the second argument is an R function that defines the transformation. The R function is applied sample-wise, expecting that the first unnamed argument is a vector of taxa counts in the same order as the phyloseq object. Additional arguments are passed on to the function specified in the second argument, providing an explicit means to include pre-computed values, previously defined parameters/thresholds, or any other object that might be appropriate for computing the transformed values of interest.

This example begins by defining a custom plot function, plot_abundance(), that uses phyloseq’s psmelt() function to define a relative abundance graphic. We will use this to compare differences in scale and distribution of the abundance values in our phyloseq object before and after transformation.



                        plot_abundance 
                        = 
                        function
                        (
                        physeq
                        ,
                        title 
                        = 
                        ""
                        ,
			     
                        Facet 
                        = 
                        "Order"
                        , 
                        Color 
                        = 
                        "Phylum"
                        ){
  
                        # Arbitrary subset, based on Phylum, for plotting
  
                        p1f 
                        = subset_taxa
                        (physeq, Phylum 
                        %in% 
                        c
                        (
                        "Firmicutes"
                        ))
  
                        mphyseq 
                        = psmelt
                        (p1f)
  mphyseq 
                        <- subset
                        (mphyseq, Abundance 
                        > 
                        0
                        )
  
                        ggplot
                        (
                        data 
                        = mphyseq, 
                        mapping 
                        = 
                        aes_string
                        (
                        x 
                        = 
                        "sex"
                        ,
                        y 
                        = 
                        "Abundance"
                        ,
                                 
                        color 
                        = Color, 
                        fill 
                        = Color))
                         +
    
                        geom_violin
                        (
                        fill 
                        = 
                        NA
                        ) 
                        +
    
                        geom_point
                        (
                        size 
                        = 
                        1
                        , 
                        alpha 
                        = 
                        0.3
                        ,
                
                        position 
                        = 
                        position_jitter
                        (
                        width 
                        = 
                        0.3
                        )) 
                        +
    
                        facet_wrap
                        (
                        facets 
                        = Facet) 
                        + 
                        scale_y_log10
                        ()
                        +
    
                        theme
                        (
                        legend.position
                        =
                        "none"
                        )
}
                    


The transformation in this case converts the counts from each sample into their frequencies, often referred to as *proportions* or *relative abundances*. This function is so simple that it is easiest to define it within the function call to transform_sample_counts().



                        # Transform to relative abundance. Save as new object.

                        ps3ra 
                        = transform_sample_counts
                        (ps3, 
                        function
                        (
                        x
                        ){x / 
                        sum
                        (x)})
                    


Now plot the abundance values before and after transformation.



                        plotBefore 
                        = plot_abundance
                        (ps3,
                        ""
                        )
plotAfter 
                        = plot_abundance
                        (ps3ra,
                        ""
                        )

                        # Combine each plot into one graphic.

                        grid.arrange
                        (
                        nrow 
                        = 
                        2
                        , plotBefore, plotAfter)
                    


**Figure 5.**
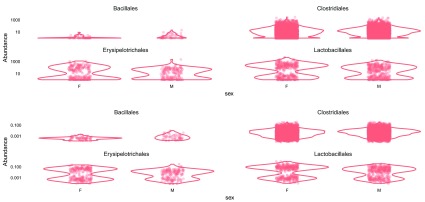
Comparison of original abundances (top panel) and relative abundances (lower).

### Subset by taxonomy

Notice on the previous plot that *Lactobacillales* appears to be a taxonomic Order with bimodal abundance profile in the data. We can check for a taxonomic explanation of this pattern by plotting just that taxonomic subset of the data. For this, we subset with the subset_taxa() function, and then specify a more precise taxonomic rank to the Facet argument of the plot_abundance function that we defined above.



                        psOrd 
                        = subset_taxa
                        (ps3ra, Order 
                        == 
                        "Lactobacillales"
                        )

                        plot_abundance
                        (psOrd, 
                        Facet 
                        = 
                        "Genus"
                        , 
                        Color 
                        = 
                        NULL
                        )
                    


**Figure 6.**
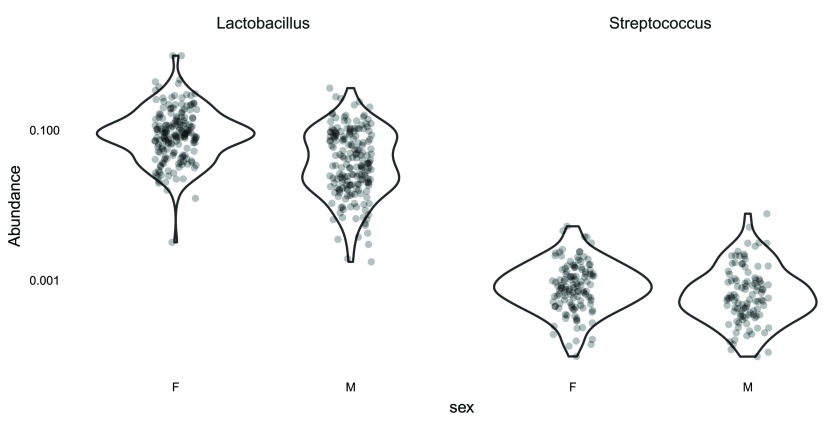
Violin plot of the relative abundances of Lactobacillales taxonomic Order, grouped by host sex and genera. Here it is clear that the apparent biomodal distribution of Lactobacillales on the previous plot was the result of a mixture of two different genera, with the typical *Lactobacillus* relative abundance much larger than *Streptococcus*.

At this stage in the workflow, after converting raw reads to interpretable species abundances, and after filtering and transforming these abundances to focus attention on scientifically meaningful quantities, we are in a position to consider more careful statistical analysis. R is an ideal environment for performing these analyses, as it has an active community of package developers building simple interfaces to sophisticated techniques. As a variety of methods are available, there is no need to commit to any rigid analysis strategy a priori. Further, the ability to easily call packages without reimplementing methods frees researchers to iterate rapidly through alternative analysis ideas. The advantage of performing this full workflow in R is that this transition from bioinformatics to statistics is effortless.

We back these claims by illustrating several analyses on the mouse data prepared above. We experiment with several flavors of exploratory ordination before shifting to more formal testing and modeling, explaining the settings in which the different points of view are most appropriate. Finally, we provide example analyses of multitable data, using a study in which both metabolomic and microbial abundance measurements were collected on the same samples, to demonstrate that the general workflow presented here can be adapted to the multitable setting.



                        .cran_packages 
                        <- c
                        (
                        "knitr"
                        , 
                        "phyloseqGraphTest"
                        , 
                        "phyloseq"
                        , 
                        "shiny"
                        ,

                                          "miniUI"
                        , 
                        "caret"
                        , 
                        "pls"
                        , 
                        "e1071"
                        , 
                        "ggplot2"
                        , 
                        "randomForest"
                        ,

                                          "vegan"
                        , 
                        "plyr"
                        , 
                        "dplyr"
                        , 
                        "ggrepel"
                        , 
                        "nlme"
                        ,

                                          "reshape2",
                        "devtools"
                        , 
                        "PMA"
                        , 
                        "structSSI"
                        , 
                        "ade4"
                        ,

                                          "igraph"
                        , 
                        "ggnetwork"
                        , 
                        "intergraph"
                        , 
                        "scales"
                        )

                        .github_packages 
                        <- c
                        (
                        "jfukuyama/phyloseqGraphTest"
                        )

                        .bioc_packages 
                        <- c
                        (
                        "phyloseq"
                        , 
                        "genefilter"
                        , 
                        "impute"
                        )


                        
# Install CRAN packages (if not already installed)

                        .inst 
                        <- 
                        .cran_packages 
                        %in% 
                        installed.packages
                        ()

                        if 
                        (
                        any
                        (!.
                        inst)){

                          install.packages
                        (.cran_packages[
                        !
                        .inst],
                        repos 
                        = 
                        "http://cran.rstudio.com/"
                        )

                        }


                        .inst 
                        <- 
                        .github_packages 
                        %in% 
                        installed.packages
                        ()

                        if 
                        (
                        any
                        (
                        !
                        .inst)){

                          devtools
                        ::
                        install_github
                        (.github_packages[
                        !
                        .inst])

                        }


                        .inst 
                        <- 
                        .bioc_packages 
                        %in% 
                        installed.packages
                        ()

                        if
                        (
                        any
                        (
                        !
                        .inst)){

                          source
                        (
                        "http://bioconductor.org/biocLite.R"
                        )

                          biocLite
                        (.bioc_packages[
                        !
                        .inst])

                        }



### Preprocessing

**Figure 7.**
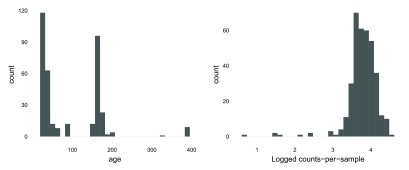
Preliminary plots suggest certain preprocessing steps. The histogram on the left motivates the creation of a new categorical variable, binning age into one of the three peaks. The histogram on the right suggests that a log (1 + *x*) transformation is sufficient for normalizing the abundance data.

Before doing the multivariate projections, we will add a few columns to our sample data, which can then be used to annotate plots. From Figure 7, we see that the ages of the mice come in a couple of groups, and so we make a categorical variable corresponding to young, middle-aged, and old mice. We also record the total number of counts seen in each sample and log-transform the data as an approximate variance stabilizing transformation.



                        qplot
                        (
                        sample_data
                        (ps)
                        $
                        age, 
                        geom 
                        = 
                        "histogram"
                        ) 
                        + 
                        xlab
                        (
                        "age"
                        )

                        qplot
                        (
                        log10
                        (
                        rowSums
                        (
                        otu_table
                        (ps)))) 
                        +
  
                        xlab
                        (
                        "Logged counts-per-sample"
                        )
                    


For a first pass, we look at principal coordinates analysis (PCoA) with either the Bray-Curtis dissimilarity on the weighted Unifrac distance. We see immediately that there are six outliers. These turn out to be the samples from females 5 and 6 on day 165 and the samples from males 3, 4, 5, and 6 on day 175. We will take them out, since we are mainly interested in the relationships between the non-outlier points.



                        pslog 
                        <- transform_sample_counts
                        (ps, 
                        function
                        (
                        x
                        ) 
                        log
                        (
                        1 
                        + 
                        x))

                        sample_data
                        (pslog)
                        $
                        age_binned 
                        <- cut
                        (
                        sample_data
                        (pslog)
                        $
                        age,
  				          
                        breaks 
                        = 
                        c
                        (
                        0
                        , 
                        100
                        , 
                        200
                        , 
                        400
                        ))
out.wuf.log 
                        <- ordinate
                        (pslog, 
                        method 
                        = 
                        "MDS"
                        , 
                        distance 
                        = 
                        "wunifrac"
                        )
                    




                        evals 
                        <- 
                        out.wuf.log
                        $
                        values
                        $
                        Eigenvalues

                        plot_ordination
                        (pslog, out.wuf.log, 
                        color 
                        = 
                        "age_binned"
                        ) 
                        +
  
                        labs
                        (
                        col 
                        = 
                        "Binned Age"
                        ) 
                        +

                          coord_fixed
                        (
                        sqrt
                        (evals[
                        2
                        ] / evals[
                        1
                        ]))
                    


**Figure 8.**
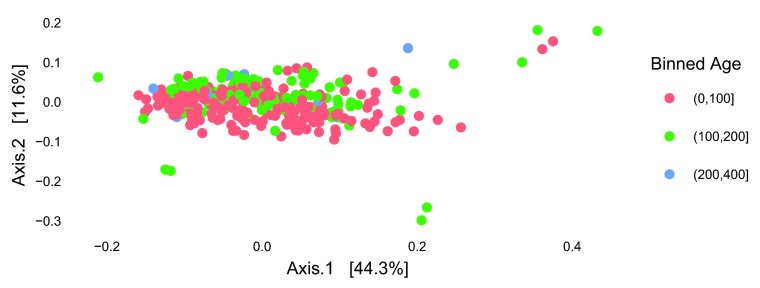
An ordination on the logged abundance data reveals a few outliers.

Before we continue, we should check the two female outliers – they have been taken over by the same OTU/RSV, which has a relative abundance of over 90% in each of them. This is the only time in the entire data set that this RSV has such a high relative abundance – the rest of the time it is below 20%. In particular, its diversity is by far the lowest of all the samples.



                        rel_abund 
                        <- t
                        (
                        apply
                        (
                        otu_table
                        (ps), 
                        1
                        , 
                        function
                        (
                        x
                        ) x / 
                        sum
                        (x)))

                        qplot
                        (rel_abund[, 
                        12
                        ], 
                        geom 
                        = 
                        "histogram"
                        ) 
                        +
  
                        xlab
                        (
                        "Relative abundance"
                        )
                    


**Figure 9.**
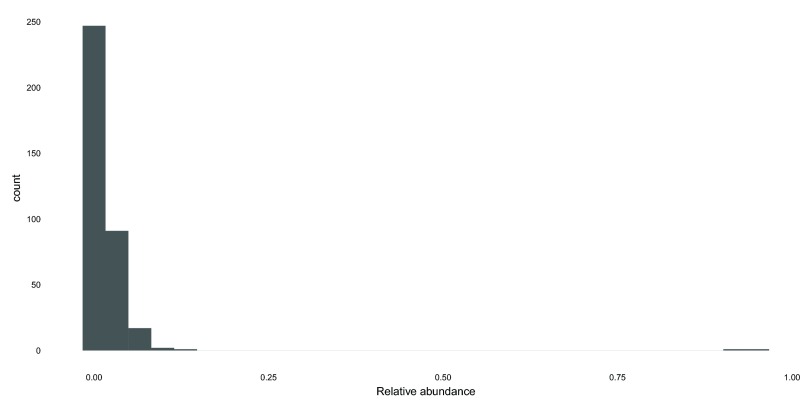
The outlier samples are dominated by a single RSV.

### Aspect ratio of ordination plots

In the ordination plots in Figure 8– Figure 14, you may have noticed as did the reviewers of the first version of the paper, that the maps are not presented as square representations as is often the case in standard PCoA and PCA plots in the literature.

The reason for this is that as we are trying to represent the distances between samples as faithfully as possible; we have to take into account that the second eigenvalue is always smaller than the first, sometimes considerably so, thus we normalize the axis norm ratios to the relevant eigenvalue ratios.

### Different ordination projections

As we have seen, an important first step in analyzing microbiome data is to do unsupervised, exploratory analysis. This is simple to do in *phyloseq*, which provides many distances and ordination methods.

After documenting the outliers, we are going to compute ordinations with these outliers removed and more carefully study the output. We see that there is a fairly substantial age effect that is consistent between all the mice, male and female, and from different litters. We’ll first perform a PCoA using Bray-Curtis dissimilarity.

The first plot shows the ordination of the samples, and we see that the second axis corresponds to an age effect, with the samples from the younger and older mice separating fairly well. The first axis correlates fairly well with library size (this is not shown). The first axis explains about twice the variability than the first, this translates into the elongated form of the ordination plot.



                        setup_example
                        (
                        c
                        (
                        "phyloseq"
                        , 
                        "ggplot2"
                        , 
                        "plyr"
                        , 
                        "dplyr"
                        , 
                        "reshape2"
                        ,
                  
                        "ade4"
                        , 
                        "ggrepel"
                        ))
out.bc.log 
                        <- ordinate
                        (pslog, 
                        method 
                        = 
                        "MDS"
                        , 
                        distance 
                        = 
                        "bray"
                        )
                    




                        evals 
                        <- 
                        out.dpcoa.log
                        $
                        eig

                        plot_ordination
                        (pslog, out.dpcoa.log, 
                        color 
                        = 
                        "age_binned"
                        ,
                  
                        shape 
                        = 
                        "family_relationship"
                        ) +
  
                        coord_fixed
                        (
                        sqrt
                        (evals[
                        2
                        ] / evals[
                        1
                        ])) 
                        +
  
                        labs
                        (
                        col 
                        = 
                        "Binned Age"
                        , 
                        shape 
                        = 
                        "Litter"
                        )
                    


**Figure 10.**
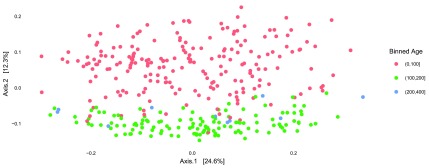
A PCoA plot using Bray-Curtis distance between samples.



                        evals 
                        <- 
                        out.bc.log
                        $
                        values
                        $
                        Eigenvalues

                        plot_ordination
                        (pslog, out.bc.log, 
                        color 
                        = 
                        "age_binned"
                        ) 
                        +
  
                        coord_fixed
                        (
                        sqrt
                        (evals[
                        2
                        ] / evals[
                        1
                        ])) 
                        +
  
                        labs
                        (
                        col 
                        = 
                        "Binned Age"
                        )
                    


**Figure 11.**
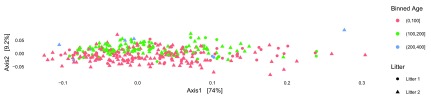
A DPCoA plot incorporates phylogenetic information, but is dominated by the first axis.

Next we look at double principal coordinates analysis (DPCoA) ^18– 20^, which is a phylogenetic ordination method and that provides a biplot representation of both samples and taxonomic categories. We see again that the second axis corresponds to young vs. old mice, and the biplot suggests an interpretation of the second axis: samples that have larger scores on the second axis have more taxa from Bacteroidetes and one subset of Firmicutes.



                        out.dpcoa.log 
                        <- ordinate
                        (pslog, 
                        method 
                        = 
                        "DPCoA"
                        )
                    


Finally, we can look at the results of PCoA with weighted Unifrac. As before, we find that the second axis is associated with an age effect, which is fairly similar to DPCoA. This is not surprising, because both are phylogenetic ordination methods taking abundance into account. However, when we compare biplots, we see that the DPCoA gave a much cleaner interpretation of the second axis, compared to weighted Unifrac.



                        out.wuf.log 
                        <- ordinate
                        (pslog, 
                        method 
                        = 
                        "PCoA"
                        , 
                        distance 
                        =
                        "wunifrac"
                        )
                    


### PCA on ranks

Microbial abundance data is often heavy-tailed, and sometimes it can be hard to identify a transformation that brings the data to normality. In these cases, it can be safer to ignore the raw abundances altogether, and work instead with ranks. We demonstrate this idea using a rank-transformed version of the data to perform PCA. First, we create a new matrix, representing the abundances by their ranks, where the microbe with the smallest in a sample gets mapped to rank 1, second smallest rank 2, etc.



                        plot_ordination
                        (pslog, out.dpcoa.log, 
                        type 
                        = 
                        "species"
                        , 
                        color 
                        = 
                        "Phylum"
                        ) 
                        +
  
                        coord_fixed
                        (
                        sqrt
                        (evals[
                        2
                        ] 
                        / 
                        evals[
                        1
                        ]))
                    


**Figure 12.**
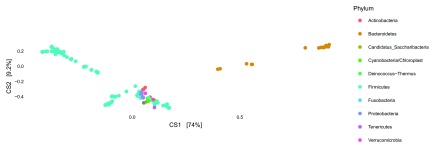
The DPCoA sample positions can be interpreted with respect to the species coordinates in this display.



                        evals 
                        <- 
                        out.wuf.log
                        $
                        values
                        $
                        Eigenvalues

                        plot_ordination
                        (pslog, out.wuf.log, 
                        color 
                        = 
                        "age_binned"
                        ,
                  
                        shape 
                        = 
                        "family_relationship"
                        ) +
  
                        coord_fixed
                        (
                        sqrt
                        (evals[
                        2
                        ] / evals[
                        1
                        ])) +
  
                        labs
                        (
                        col 
                        = 
                        "Binned Age"
                        , 
                        shape 
                        = 
                        "Litter"
                        )
                    


**Figure 13.**
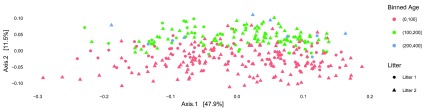
The sample positions produced by a PCoA using weighted Unifrac.



                        plot_ordination
                        (pslog, out.wuf.log, 
                        type 
                        = 
                        "species"
                        , 
                        color 
                        = 
                        "Phylum"
                        ) 
                        +
  
                        coord_fixed
                        (
                        sqrt
                        (evals[
                        2
                        ] / evals[
                        1
                        ]))
                    


**Figure 14.**
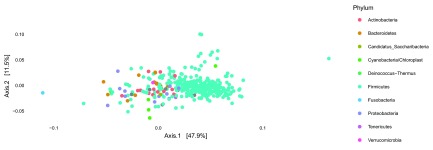
Species coordinates that can be used to interpret the sample positions from PCoA with weighted Unifrac. Compared to the representation in Figure 12, this display is harder to interpret.



                        abund 
                        <- otu_table
                        (pslog)

                        abund_ranks 
                        <- t
                        (
                        apply
                        (abund, 
                        1
                        , rank))
                    


**Figure 15.**
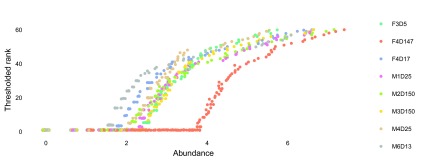
The association between abundance and rank, for a few randomly selected samples. The numbers of the *y*-axis are those supplied to PCA.

Naively using these ranks could make differences between pairs of low and high abundance microbes comparable. In the case where many bacteria are absent or present at trace amounts, an artificially large difference in rank could occur ^21^ for minimally abundant taxa. To avoid this, all those microbes with rank below some threshold are set to be tied at 1. The ranks for the other microbes are shifted down, so there is no large gap between ranks. This transformation is illustrated in Figure 15.



                        abund_ranks 
                        <- 
                        abund_ranks - 
                        329

                        abund_ranks[abund_ranks 
                        < 
                        1
                        ] 
                        <- 
                        1
                    


**Figure 16.**
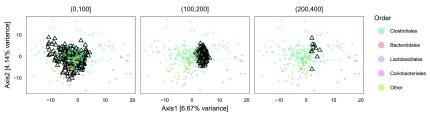
The biplot resulting from the PCA after the truncated-ranking transformation.

We can now perform PCA and study the resulting biplot, given in Figure 16. To produce annotation for this figure, we used the following block.



                        ranks_pca 
                        <- dudi.pca
                        (abund_ranks, 
                        scannf 
                        = F, 
                        nf 
                        = 
                        3
                        )
row_scores 
                        <- data.frame
                        (
                        li 
                        = ranks_pca
                        $
                        li,
                            
                        SampleID 
                        = 
                        rownames
                        (abund_ranks))
col_scores 
                        <- data.frame
                        (
                        co 
                        = ranks_pca
                        $
                        co,
                            
                        seq 
                        = 
                        colnames
                        (abund_ranks))

tax 
                        <- tax_table
                        (ps)
                        @
                        .Data 
                        %>%
  
                        data.frame
                        (
                        stringsAsFactors 
                        = 
                        FALSE
                        )
tax
                        $
                        seq 
                        <- rownames
                        (tax)


                        main_orders 
                        <- c
                        (
                        "Clostridiales"
                        , 
                        "Bacteroidales"
                        , 
                        "Lactobacillales"
                        ,
                   
                        "Coriobacteriales"
                        )
tax
                        $
                        Order[
                        !
                        (tax
                        $
                        Order 
                        %in% 
                        main_orders)] 
                        <- 
                        "Other"

                        tax
                        $
                        Order 
                        <- factor
                        (tax
                        $
                        Order, 
                        levels 
                        = 
                        c
                        (main_orders, 
                        "Other"
                        ))
tax
                        $
                        otu_id 
                        <- seq_len
                        (
                        ncol
                        (
                        otu_table
                        (ps)))

row_scores 
                        <- 
                        row_scores 
                        %>%
  
                        left_join
                        (
                        sample_data
                        (pslog))
col_scores 
                        <- 
                        col_scores 
                        %>%
  
                        left_join
                        (tax)
                    


The results are similar to the PCoA analyses computed without applying a truncated-ranking transformation, reinforcing our confidence in the analysis on the original data.



                        abund_df 
                        <- 
                        melt
                        (abund, 
                        value.name 
                        = 
                        "abund"
                        ) 
                        %>%
  
                        left_join
                        (
                        melt
                        (abund_ranks, 
                        value.name 
                        = 
                        "rank"
                        ))

                        colnames
                        (abund_df) 
                        <- 
                        c
                        (
                        "sample"
                        , 
                        "seq"
                        , 
                        "abund"
                        , 
                        "rank"
                        )

abund_df 
                        <- 
                        melt
                        (abund, 
                        value.name 
                        = 
                        "abund"
                        ) 
                        %>%
  
                        left_join
                        (
                        melt
                        (abund_ranks, 
                        value.name 
                        = 
                        "rank"
                        ))

                        colnames
                        (abund_df) 
                        <- 
                        c
                        (
                        "sample"
                        , 
                        "seq"
                        , 
                        "abund"
                        , 
                        "rank"
                        )

sample_ix 
                        <- 
                        sample
                        (
                        1
                        :
                        nrow
                        (abund_df), 
                        8
                        )

                        ggplot
                        (abund_df 
                        %>%
          
                        filter
                        (sample 
                        %in% 
                        abund_df
                        $
                        sample[sample_ix])) 
                        +
  
                        geom_point
                        (
                        aes
                        (
                        x 
                        = abund, 
                        y 
                        = rank, 
                        col 
                        = sample),
              
                        position 
                        = 
                        position_jitter
                        (
                        width 
                        = 
                        0.2
                        ), 
                        size 
                        = 
                        .7
                        ) 
                        +
  
                        labs
                        (
                        x 
                        = 
                        "Abundance"
                        , 
                        y 
                        = 
                        "Thresholded rank"
                        ) 
                        +
  
                        scale_color_brewer
                        (
                        palette 
                        = 
                        "Set2"
                        )
                    


### Canonical correspondence

Canonical Correspondence Analysis (CCpnA) is an approach to ordination of a species by sample table that incorporates supplemental information about the samples. As before, the purpose of creating biplots is to determine which types of bacterial communities are most prominent in different mouse sample types. It can be easier to interpret these biplots when the ordering between samples reflects sample characteristics – variations in age or litter status in the mouse data, for example – and this central to the design of CCpnA.

The function allows to create biplots where the positions of samples are determined by similarity in both species signatures and environmental characteristics; in contrast, principal components analysis or correspondence analysis only look at species signatures. More formally, it ensures that the resulting CCpnA directions lie in the span of the environmental variables; thorough treatments are available in 22, 23.

Like PCoA and DPCoA, this method can be run using ordinate in *phyloseq*. In order to use supplemental sample data, it is necessary to provide an extra argument, specifying which of the features to consider – otherwise, *phyloseq* defaults to using all sample_data measurements when producing the ordination.



                        ps_ccpna 
                        <- ordinate
                        (pslog, 
                        "CCA"
                        , 
                        formula 
                        = pslog ~ age_binned + family_relationship)
                    


To access the positions for the biplot, we can use the scores function in the *vegan*. Further, to facilitate figure annotation, we also join the site scores with the environmental data in the sample_data slot. Of the 23 total taxonomic orders, we only explicitly annotate the four most abundant – this makes the biplot easier to read.



                        ps_scores 
                        <- 
                        vegan
                        ::
                        scores
                        (ps_ccpna)
sites 
                        <- data.frame
                        (ps_scores
                        $
                        sites)
sites
                        $
                        SampleID 
                        <- rownames
                        (sites)
sites 
                        <- 
                        sites 
                        %>%
  
                        left_join
                        (
                        sample_data
                        (ps))

species 
                        <- data.frame
                        (ps_scores
                        $
                        species)
species
                        $
                        otu_id 
                        <- seq_along
                        (
                        colnames
                        (
                        otu_table
                        (ps)))
species 
                        <- 
                        species 
                        %>%
  
                        left_join
                        (tax)
                    




                        evals_prop 
                        <- 
                        100 
                        * 
                        (ranks_pca
                        $
                        eig / 
                        sum
                        (ranks_pca
                        $
                        eig))

                        ggplot
                        () 
                        +
  
                        geom_point
                        (
                        data 
                        = row_scores, 
                        aes
                        (
                        x 
                        = li.Axis1, 
                        y 
                        = li.Axis2), 
                        shape 
                        = 
                        2
                        ) 
                        +
  
                        geom_point
                        (
                        data 
                        = col_scores, 
                        aes
                        (
                        x 
                        = 
                        25 
                        * 
                        co.Comp1, 
                        y 
                        = 
                        25 
                        * 
                        co.Comp2, 
                        col 
                        = Order),
              
                        size 
                        = 
                        .3
                        , 
                        alpha 
                        = 
                        0.6
                        ) 
                        +
  
                        scale_color_brewer
                        (
                        palette 
                        = 
                        "Set2"
                        ) 
                        +
  
                        facet_grid
                        (~ age_binned) 
                        +
  
                        guides
                        (
                        col 
                        = 
                        guide_legend
                        (
                        override.aes 
                        = 
                        list
                        (
                        size 
                        = 
                        3
                        ))) 
                        +
  
                        labs
                        (
                        x 
                        = 
                        sprintf
                        (
                        "Axis1 [%s%% variance]"
                        , 
                        round
                        (evals_prop[
                        1
                        ], 
                        2
                        )), 

                               y 
                        = 
                        sprintf
                        (
                        "Axis2 [%s%% variance]"
                        , 
                        round
                        (evals_prop[
                        2
                        ], 
                        2
                        ))) 
                        +
  
                        coord_fixed
                        (
                        sqrt
                        (ranks_pca
                        $
                        eig[
                        2
                        ] / ranks_pca
                        $
                        eig[
                        1
                        ])) 
                        +
  
                        theme
                        (
                        panel.border 
                        = 
                        element_rect
                        (
                        color 
                        = 
                        "#787878"
                        , 
                        fill 
                        = 
                        alpha
                        (
                        "white"
                        , 
                        0
                        )))
                    


**Figure 17.**
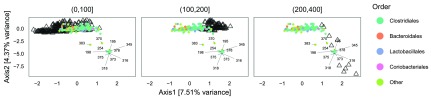
The mouse and bacteria scores generated by CCpnA. The sites and species are triangles and circles, respectively. The separate panels indicate different age groups.



**Figure 18.**
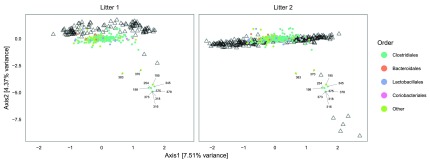
The analogue to Figure 17, faceting by litter membership rather than age bin.

Figure 17 and Figure 18 plot these annotated scores, splitting sites by their age bin and litter membership, respectively. We have labeled individual microbes that are outliers along the second CCpnA direction.

Evidently, the first CCpnA direction distinguishes between mice in the two main age bins. Circles on the left and right of the biplot represent microbes that are characteristic of younger and older mice, respectively. The second CCpnA direction splits off the few mice in the oldest age group; it also partially distinguishes between the two litters. These samples low in the second CCpnA direction have more of the outlier microbes than the others.

This CCpnA analysis supports our conclusions from the earlier ordinations – the main difference between the microbiome communities of the different mice lies along the age axis. However, in situations where the influence of environmental variables is not so strong, CCA can have more power in detecting such associations. In general, it can be applied whenever it is desirable to incorporate supplemental data, but in a way that (1) is less aggressive than supervised methods, and (2) can use several environmental variables at once.



                        evals_prop 
                        <- 
                        100 
                        * 
                        ps_ccpna
                        $
                        CCA
                        $
                        eig[
                        1
                        :
                        2
                        ] / 
                        sum
                        (ps_ccpna
                        $
                        CA
                        $
                        eig)

                        ggplot
                        () 
                        +
  
                        geom_point
                        (
                        data 
                        = sites, 
                        aes
                        (
                        x 
                        = CCA1, 
                        y 
                        = CCA2), 
                        shape 
                        = 
                        2
                        , 
                        alpha 
                        = 
                        0.5
                        ) 
                        +
  
                        geom_point
                        (
                        data 
                        = species, 
                        aes
                        (
                        x 
                        = CCA1, 
                        y 
                        = CCA2, 
                        col 
                        = Order), 
                        size 
                        = 
                        0.5
                        ) 
                        +
  
                        geom_text_repel
                        (
                        data 
                        = species 
                        %>% 
                        filter
                        (CCA2 
                        < -
                        2
                        ),
		    
                        aes
                        (
                        x 
                        = CCA1, 
                        y 
                        = CCA2, 
                        label 
                        = otu_id),
		    
                        size 
                        = 
                        1.5
                        , 
                        segment.size 
                        = 
                        0.1
                        ) 
                        +
  
                        facet_grid
                        (. ~ age_binned) 
                        +
  
                        guides
                        (
                        col 
                        = 
                        guide_legend
                        (
                        override.aes 
                        = 
                        list
                        (
                        size 
                        = 
                        3
                        ))) 
                        +
  
                        labs
                        (
                        x 
                        = 
                        sprintf
                        (
                        "Axis1 [%s%% variance]"
                        , 
                        round
                        (evals_prop[
                        1
                        ], 
                        2
                        )), 

                               y 
                        = 
                        sprintf
                        (
                        "Axis2 [%s%% variance]"
                        , 
                        round
                        (evals_prop[
                        2
                        ], 
                        2
                        ))) 
                        +
  
                        scale_color_brewer
                        (
                        palette 
                        = 
                        "Set2"
                        ) 
                        +
  
                        coord_fixed
                        (
                        sqrt
                        (ps_ccpna
                        $
                        CCA
                        $
                        eig[
                        2
                        ] / ps_ccpna
                        $
                        CCA
                        $
                        eig[
                        1
                        ])
                        *
                        0.33
                        ) 
                        +
  
                        theme
                        (
                        panel.border 
                        = 
                        element_rect
                        (
                        color 
                        = 
                        "#787878"
                        , 
                        fill 
                        = 
                        alpha
                        (
                        "white"
                        , 
                        0
                        )))
                    


                        ggplot
                        () 
                        +
  
                        geom_point
                        (
                        data 
                        = sites, 
                        aes
                        (
                        x 
                        = CCA1
                        , 
                        y 
                        = CCA2
                        ), 
                        shape 
                        = 
                        2
                        , 
                        alpha 
                        = 
                        0.5
                        ) 
                        +
  
                        geom_point
                        (
                        data 
                        = species, 
                        aes
                        (
                        x 
                        = CCA1, 
                        y 
                        = CCA2, 
                        col 
                        = Order), 
                        size 
                        = 
                        0.5
                        ) 
                        +
  
                        geom_text_repel
                        (
                        data 
                        = species 
                        %>% 
                        filter
                        (CCA2 
                        < -
                        2
                        ),
                    
                        aes
                        (
                        x 
                        = CCA1, 
                        y 
                        = CCA2, 
                        label 
                        = otu_id),
		    
                        size 
                        = 
                        1.5
                        , 
                        segment.size 
                        = 
                        0.1
                        ) 
                        +
  
                        facet_grid
                        (. ~ family_relationship) 
                        +
  
                        guides
                        (
                        col 
                        = 
                        guide_legend
                        (
                        override.aes 
                        = 
                        list
                        (
                        size 
                        = 
                        3
                        ))) 
                        +
  
                        labs
                        (
                        x 
                        = 
                        sprintf
                        (
                        "Axis1 [%s%% variance]"
                        , 
                        round
                        (evals_prop[
                        1
                        ], 
                        2
                        )),
        
                        y 
                        = 
                        sprintf
                        (
                        "Axis2 [%s%% variance]"
                        , 
                        round
                        (evals_prop[
                        2
                        ], 
                        2
                        ))) 
                        +
  
                        scale_color_brewer
                        (
                        palette 
                        = 
                        "Set2"
                        ) 
                        +
  
                        coord_fixed
                        (
                        sqrt
                        (ps_ccpna
                        $
                        CCA
                        $
                        eig[
                        2
                        ] / ps_ccpna
                        $
                        CCA
                        $
                        eig[
                        1
                        ])
                        *
                        0.45   
                        ) 
                        +
  
                        theme
                        (
                        panel.border 
                        = 
                        element_rect
                        (
                        color 
                        = 
                        "#787878"
                        , 
                        fill 
                        = 
                        alpha
                        (
                        "white"
                        , 
                        0
                        )))
                    


### Supervised learning

Here we illustrate some supervised learning methods that can be easily run in R. The *caret* package wraps many prediction algorithms available in R and performs parameter tuning automatically. Since we saw that microbiome signatures change with age, we’ll apply supervised techniques to try to predict age from microbiome composition.

We’ll first look at Partial Least Squares (PLS) ^24^. The first step is to divide the data into training and test sets, with assignments done by mouse, rather than by sample, to ensure that the test set realistically simulates the collection of new data. Once we split the data, we can use the train function to fit the PLS model.



                        setup_example
                        (
                        c
                        (
                        "phyloseq"
                        , 
                        "ggplot2"
                        , 
                        "caret"
                        , 
                        "plyr"
                        , 
                        "dplyr"
                        ))

                        sample_data
                        (pslog)
                        $
                        age2 
                        <- cut
                        (
                        sample_data
                        (pslog)
                        $
                        age, 
                        c
                        (
                        0
                        , 
                        100
                        , 
                        400
                        ))
dataMatrix 
                        <- data.frame
                        (
                        age 
                        = 
                        sample_data
                        (pslog)
                        $
                        age2, 
                        otu_table
                        (pslog))

                        # take 8 mice at random to be the training set, and the remaining 4 the test set

                        trainingMice 
                        <- sample
                        (
                        unique
                        (
                        sample_data
                        (pslog)
                        $
                        host_subject_id), 
                        size 
                        = 
                        8
                        )
inTrain 
                        <- which
                        (
                        sample_data
                        (pslog)
                        $
                        host_subject_id 
                        %in% 
                        trainingMice)
training 
                        <- 
                        dataMatrix[inTrain,]
testing 
                        <- 
                        dataMatrix[-inTrain,]
plsFit 
                        <- train
                        (age 
                        ~ 
                        ., 
                        data 
                        = training,
		  
                        method 
                        = 
                        "pls"
                        , 
                        preProc 
                        = 
                        "center"
                        )
                    


Next we can predict class labels on the test set using the predict function and compare to the truth. We see that the method does an excellent job of predicting age.



                        plsClasses 
                        <- predict
                        (plsFit, 
                        newdata 
                        = testing)

                        table
                        (plsClasses, testing
                        $
                        age)

##
## plsClasses (0,100] (100,400]
##   (0,100] 	   64 	      0
##   (100,400] 	    2 	     46
                    


As another example, we can try out random forests. This is run in exactly the same way as PLS, by switching the method argument from pls to rf. Random forests also perform well at the prediction task on this test set, though there are more old mice misclassified as young.



                        rfFit 
                        <- train
                        (age 
                        ~ 
                        ., 
                        data 
                        = training, 
                        method 
                        = 
                        "rf"
                        ,
 		 
                        preProc 
                        = 
                        "center"
                        , 
                        proximity 
                        = 
                        TRUE
                        )
rfClasses 
                        <- predict
                        (rfFit, 
                        newdata 
                        = testing)

                        table
                        (rfClasses, testing
                        $
                        age)

##
## rfClasses (0,100] (100,400]
##   (0,100] 	  65 	     7
##   (100,400] 	   1 	    39
                    


To interpret these PLS and random forest results, it is standard to produce biplots and proximity plots, respectively. The code below extracts coordinates and supplies annotation for points to include on the PLS biplot.



                        pls_biplot 
                        <- list
                        (
                        "loadings" 
                        = 
                        loadings
                        (plsFit
                        $
                        finalModel),
		     
                        "scores" 
                        = 
                        scores
                        (plsFit
                        $
                        finalModel))

                        class
                        (pls_biplot
                        $
                        scores) 
                        <- 
                        "matrix"


                        pls_biplot
                        $
                        scores 
                        <- data.frame
                        (
                        sample_data
                        (pslog)[inTrain, ],
				pls_biplot
                        $
                        scores)
                    




                        tax 
                        <- tax_table
                        (ps)
                        @
                        .Data 
                        %>%
  
                        data.frame
                        (
                        stringsAsFactors 
                        = 
                        FALSE
                        )
main_orders 
                        <- c
                        (
                        "Clostridiales"
                        , 
                        "Bacteroidales"
                        , 
                        "Lactobacillales"
                        ,
		   
                        "Coriobacteriales"
                        )
tax
                        $
                        Order[
                        !
                        (tax
                        $
                        Order 
                        %in% 
                        main_orders)] 
                        <- 
                        "Other"

                        tax
                        $
                        Order 
                        <- factor
                        (tax
                        $
                        Order, 
                        levels 
                        = 
                        c
                        (main_orders, 
                        "Other"
                        ))

                        class
                        (pls_biplot
                        $
                        loadings) 
                        <- 
                        "matrix"

                        pls_biplot
                        $
                        loadings 
                        <- data.frame
                        (tax, pls_biplot
                        $
                        loadings)
                    


**Figure 19.**
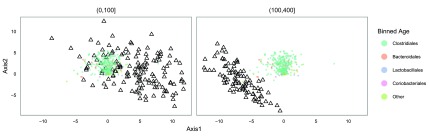
PLS produces a biplot representation designed to separate samples by a response variable.

The resulting biplot is displayed in Figure 19; it can be interpreted similarly to earlier ordination diagrams, with the exception that the projection is chosen with an explicit reference to the binned age variable. Specifically, PLS identifies a subspace to maximize discrimination between classes, and the biplot displays sample projections and RSV coefficients with respect to this subspace.



                        ggplot
                        () 
                        +
  
                        geom_point
                        (
                        data 
                        = pls_biplot
                        $
                        scores,
	      
                        aes
                        (
                        x 
                        = Comp.1, 
                        y 
                        = Comp.2), 
                        shape 
                        = 
                        2
                        ) 
                        +
  
                        geom_point
                        (
                        data 
                        = pls_biplot
                        $
                        loadings,
	      
                        aes
                        (
                        x 
                        = 
                        25 
                        * 
                        Comp.1, 
                        y 
                        = 
                        25 
                        * 
                        Comp.2, 
                        col 
                        = Order),
	      
                        size 
                        = 
                        0.3
                        , 
                        alpha 
                        = 
                        0.6
                        ) 
                        +
  
                        scale_color_brewer
                        (
                        palette 
                        = 
                        "Set2"
                        ) 
                        +
  
                        labs
                        (
                        x 
                        = 
                        "Axis1"
                        , 
                        y 
                        = 
                        "Axis2"
                        , 
                        col 
                        = 
                        "Binned Age"
                        ) 
                        +
  
                        guides
                        (
                        col 
                        = 
                        guide_legend
                        (
                        override.aes 
                        = 
                        list
                        (
                        size 
                        = 
                        3
                        ))) 
                        +
  
                        facet_grid
                        ( 
                        ~ 
                        age2) 
                        +
  
                        theme
                        (
                        panel.border 
                        = 
                        element_rect
                        (
                        color 
                        = 
                        "#787878"
                        , 
                        fill 
                        = 
                        alpha
                        (
                        "white"
                        , 
                        0
                        )))
                    


**Figure 20.**
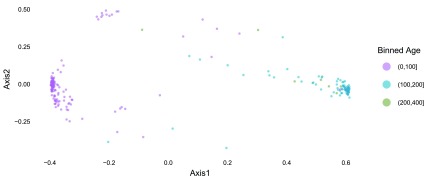
The random forest model determines a distance between samples, which can be input into PCoA to produce a proximity plot.

A random forest proximity plot is displayed in Figure 20. To generate this representation, a distance is calculated between samples based on how frequently sample occur in the same tree partition in the random forest’s bootstrapping procedure. If a pair of samples frequently occur in the same partition, the pair is assigned a low distance. The resulting distances are then input to PCoA, giving a glimpse into the random forests’ otherwise complex classification mechanism. The separation between classes is clear, and manually inspecting points would reveal what types of samples are easier or harder to classify.



                        rf_prox 
                        <- cmdscale
                        (
                        1 
                        - 
                        rfFit
                        $
                        finalModel
                        $
                        proximity) 
                        %>%
  
                        data.frame
                        (
                        sample_data
                        (pslog)[inTrain, ])
                    




                        ggplot
                        (rf_prox) 
                        +
  
                        geom_point
                        (
                        aes
                        (
                        x 
                        = X1, 
                        y 
                        = X2, 
                        col 
                        = age_binned),
              
                        size 
                        = 
                        .4
                        , 
                        alpha 
                        = 
                        0.6
                        ) 
                        +
  
                        scale_color_manual
                        (
                        values 
                        = 
                        c
                        (
                        "#A66EB8"
                        , 
                        "#238DB5"
                        , 
                        "#748B4F"
                        )) 
                        +
  
                        guides
                        (
                        col 
                        = 
                        guide_legend
                        (
                        override.aes 
                        = 
                        list
                        (
                        size 
                        = 
                        3
                        ))) 
                        +
  
                        labs
                        (
                        col 
                        = 
                        "Binned Age"
                        , 
                        x 
                        = 
                        "Axis1"
                        , 
                        y 
                        = 
                        "Axis2"
                        )
                    


**Figure 21.**
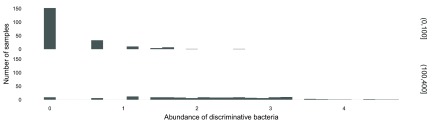
A bacteria in genus *Roseburia* becomes much more abundant in the 100 to 400 day bin.

To further understand the fitted random forest model, we identify the microbe with the most influence in the random forest prediction. This turns out to be a microbe in family *Lachnospiraceae* and genus *Roseburia*. Figure 21 plots its abundance across samples; we see that it is uniformly very low from age 0 to 100 days and much higher from age 100 to 400 days.



                        as.vector
                        (
                        tax_table
                        (ps)[
                        which.max
                        (
                        importance
                        (rfFit
                        $
                        finalModel)), 
                        c
                        (
                        "Family"
                        , 
                        "Genus"
                        )])

## [1] "Lachnospiraceae" NA

impOtu 
                        <- as.vector
                        (
                        otu_table
                        (pslog)[,
                        which.max
                        (
                        importance
                        (rfFit
                        $
                        finalModel))])
maxImpDF 
                        <- data.frame
                        (
                        sample_data
                        (pslog), 
                        abund 
                        = impOtu)

                        ggplot
                        (maxImpDF) 
                        + 
                        geom_histogram
                        (
                        aes
                        (
                        x 
                        = abund)) 
                        +
  
                        facet_grid
                        (age2 
                        ~ 
                        .) 
                        +
  
                        labs
                        (
                        x 
                        = 
                        "Abundance of discriminative bacteria"
                        , 
                        y 
                        = 
                        "Number of samples"
                        )
                    


## Graph-based visualization and testing

### Creating and plotting graphs

**Figure 22.**
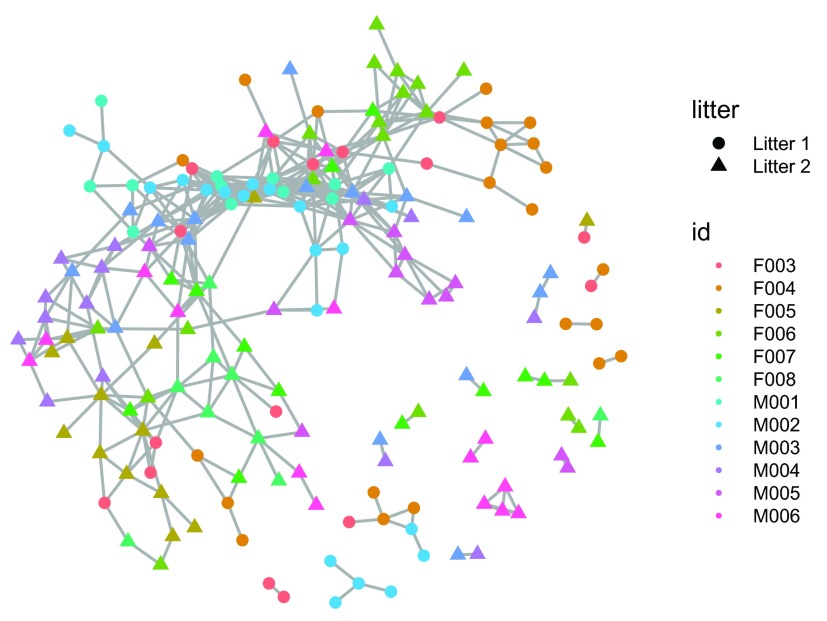
A network created by thresholding the Jaccard dissimilarity matrix. The colors represent which mouse the sample came from and the shape represents which litter the mouse was in.

Phyloseq has functionality for creating graphs based on thresholding a distance matrix, and the resulting networks can be plotting using the *ggnetwork*. This package overloads the ggplot syntax, so you can use the function ggplot on an igraph object and add geom_edges and geom_nodes geoms to plot the network. To be able to color the nodes or edges a certain way, we need to add these attributes to the igraph object. Below we create a network by thresholding the Jaccard dissimilarity (the default distance for the function make_network) at .35, and then we add an attribute to the vertices indicating which mouse the sample came from and which litter the mouse was in. Then we can plot the network with the coloring by mouse and shape by litter. We see the resulting network in Figure 22, and we can see that there is grouping of the samples by both mouse and litter.



                        setup_example
                        (
                        c
                        (
                        "igraph"
                        , 
                        "phyloseq"
                        , 
                        "phyloseqGraphTest"
                        , 
                        "ggnetwork"
                        , 
                        "intergraph"
                        ,
                        "gridExtra"
                        ))
                    




                        net 
                        <- make_network
                        (ps, 
                        max.dist
                        =
                        0.35
                        )
sampledata 
                        <- data.frame
                        (
                        sample_data
                        (ps))

                        V
                        (net)$
                        id 
                        <- 
                        sampledata[
                        names
                        (
                        V
                        (net)), 
                        "host_subject_id"
                        ]

                        V
                        (net)$
                        litter 
                        <- 
                        sampledata[
                        names
                        (
                        V
                        (net)), 
                        "family_relationship"
                        ]
                    




                        ggplot
                        (net, 
                        aes(
                        x 
                        = x, 
                        y 
                        = y, 
                        xend 
                        = xend, 
                        yend 
                        = yend), 
                        layout 
                        = 
                        "fruchtermanreingold"
                        ) 
                        +
  
                        geom_edges
                        (
                        color 
                        = 
                        "darkgray") 
                        +
  
                        geom_nodes(
                        aes(
                        color 
                        = id, 
                        shape 
                        = litter)) 
                        +
  
                        theme
                        (
                        axis.text 
                        = 
                        element_blank
                        (), 
                        axis.title 
                        = 
                        element_blank
                        (),
	 
                        legend.key.height 
                        = 
                        unit
                        (
                        0.5
                        ,
                        "line"
                        )) 
                        +
  
                        guides
                        (
                        col 
                        = 
                        guide_legend
                        (
                        override.aes 
                        = 
                        list
                        (
                        size 
                        = 
                        .25
                        )))
                    


### Graph-based two-sample tests

Graph-based two-sample tests were introduced by Friedman and Rafsky ^25^ as a generalization of the Wald-Wolfowitz runs test. They proposed the use of a minimum spanning tree (MST) based on the distances between the samples, and then counting the number of edges on the tree that were between samples in different groups. It is not necessary to use a minimum spanning tree (MST), the graph made by linking nearest neighbors ^26^ or distance thresholding can also be used as the input graph. No matter what graph we build between the samples, we can approximate a null distribution by permuting the labels of the nodes of the graph.

### Minimum Spanning Tree (MST)

We first perform a test using an MST with Jaccard dissimilarity. We want to know whether the two litters ( family_relationship) come from the same distribution. Since there is a grouping in the data by individual ( host_subject_id), we can’t simply permute all the labels, we need to maintain this nested structure – this is what the grouping argument does. Here we permute the family_relationship labels but keep the host_subject_id structure intact.

**Figure 23.**
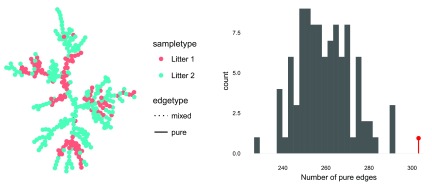
The graph and permutation histogram obtained from the minimal spanning tree with Jaccard similarity.

This test has a small *p*-value, and we reject the null hypothesis that the two samples come from the same distribution. From the plot of the minimum spanning tree in Figure 23, we see by eye that the samples group by litter more than we would expect by chance.



                        gt 
                        <- graph_perm_test
                        (ps, 
                        "family_relationship"
                        , 
                        grouping 
                        = 
                        "host_subject_id"
                        ,
			 
                        distance 
                        = 
                        "jaccard"
                        , 
                        type 
                        = 
                        "mst"
                        )
gt
                        $
                        pval
                    




                        ## [1] 0.01
                    




                        plotNet1
                        =plot_test_network
                        (gt) 
                        + 
                        theme
                        (
                        legend.text 
                        = 
                        element_text
                        (
                        size 
                        = 
                        8
                        ),
         
                        legend.title 
                        = 
                        element_text
                        (
                        size 
                        = 
                        9
                        ))

                        plotPerm1
                        =plot_permutations
                        (gt)

                        grid.arrange
                        (
                        ncol
                         = 
                        2
                        , plotNet1, plotPerm1)
                    


### Nearest neighbors

**Figure 24.**
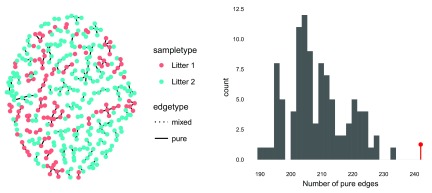
The graph and permutation histogram obtained from a nearest-neighbor graph with Jaccard similarity.

The *k*-nearest neighbors graph is obtained by putting an edge between two samples whenever one of them is in the set of *k*-nearest neighbors of the other. We see from Figure 24 that if a pair of samples has an edge between them in the nearest neighbor graph, they are overwhelmingly likely to be in the same litter.



                        gt 
                        <- graph_perm_test
                        (ps, 
                        "family_relationship"
                        , 
                        grouping 
                        = 
                        "host_subject_id"
                        ,
			 
                        distance 
                        = 
                        "jaccard"
                        , 
                        type 
                        = 
                        "knn"
                        , 
                        knn 
                        = 
                        1
                        )
                    




                        plotNet2
                        =plot_test_network
                        (gt) 
                        + 
                        theme
                        (
                        legend.text 
                        = 
                        element_text
                        (
                        size 
                        = 
                        8
                        ),
         
                        legend.title 
                        = 
                        element_text
                        (
                        size 
                        = 
                        9
                        ))

                        plotPerm2
                        =plot_permutations
                        (gt)

                        grid.arrange
                        (
                        ncol
                         = 
                        2
                        , plotNet2, plotPerm2)
                    


We can also compute the analogous test with two-nearest neighbors and the Bray-Curtis dissimilarity. The results are not shown, but the code is given below.



                        gt 
                        <- graph_perm_test
                        (ps, 
                        "family_relationship"
                        ,
                         
                        grouping 
                        = 
                        "host_subject_id"
                        ,
                         
                        distance 
                        = 
                        "bray"
                        , 
                        type 
                        = 
                        "knn"
                        , 
                        knn 
                        = 
                        2
                        )
                    


### Distance threshold

Another way of making a graph between samples is to threshold the distance matrix, this is called a geometric graph ^27^. The testing function lets the user supply an absolute distance threshold; alternatively, it can find a distance threshold such that there are a prespecified number of edges in the graph. Below we use a distance threshold so that there are 720 edges in the graph, or twice as many edges as there are samples. Heuristically, the graph we obtain isn’t as good, because there are many singletons. This reduces power, and so if the thresholded graph has this many singletons it is better to either modify the threshold or consider a MST or k-nearest neighbors graph.



                        gt 
                        <- graph_perm_test
                        (ps, 
                        "family_relationship"
                        , 
                        grouping 
                        = 
                        "host_subject_id"
                        ,
			 
                        distance 
                        = 
                        "bray"
                        , 
                        type 
                        = 
                        "threshold.nedges"
                        , 
                        nedges 
                        = 
                        720
                        ,
			 
                        keep.isolates 
                        = 
                        FALSE
                        )
                    




                        plotNet3
                        = plot_test_network
                        (gt) 
                        + 
                        theme
                        (
                        legend.text 
                        = 
                        element_text
                        (
                        size 
                        = 
                        8
                        ),
	 
                        legend.title 
                        = 
                        element_text
                        (
                        size 
                        = 
                        9
                        ))

                        plotPerm3
                        =plot_permutations
                        (gt)

                        grid.arrange
                        (
                        ncol 
                        = 
                        2
                        , plotNet3, plotPerm3)
                    


**Figure 25.**
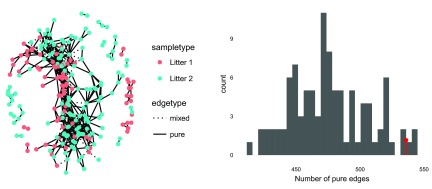
Testing using a Bray-Curtis distance thresholded graph.

Then we can try a similar procedure with an increased number of edges to see what happens (code given below but output not shown).



                        gt 
                        <- graph_perm_test
                        (ps, 
                        "family_relationship"
                        , 
                        grouping 
                        = 
                        "host_subject_id"
                        ,
			 
                        distance 
                        = 
                        "bray"
                        , 
                        type 
                        = 
                        "threshold.nedges"
                        , 
                        nedges 
                        = 
                        2000
                        ,
			 
                        keep.isolates 
                        = 
                        FALSE
                        )
                    


### Linear modeling

It is often of interest to evaluate the degree to which microbial community diversity reflects characteristics of the environment from which it was sampled. Unlike ordination, the purpose of this analysis is not to develop a representation of many bacteria with respect to sample characteristics; rather, it is to describe how a single measure of overall community structure (In particular, it need not be limited to diversity – defining univariate measures of community stability is also common, for example.) is associated with sample characteristics. This is a somewhat simpler statistical goal, and can be addressed through linear modeling, for which there are a range of approaches in R. As an example, we will used a mixed-effects model to study the relationship between mouse microbial community diversity and the age and litter variables that have been our focus so far. This choice was motivated by the observation that younger mice have noticeably lower Shannon diversities, but that different mice have different baseline diversities. The mixed-effects model is a starting point for formalizing this observation.

We first compute the Shannon diversity associated with each sample and join it with sample annotation.



                        setup_example
                        (
                        c
                        (
                        "phyloseq"
                        , 
                        "ggplot2"
                        , 
                        "nlme"
                        , 
                        "dplyr"
                        , 
                        "vegan"
                        , 
                        "reshape2"
                        ))
ps_alpha_div 
                        <- estimate_richness
                        (ps, 
                        split 
                        = 
                        TRUE
                        , 
                        measure 
                        = 
                        "Shannon"
                        )
ps_alpha_div
                        $
                        SampleID 
                        <- rownames
                        (ps_alpha_div) 
                        %>%
  
                        as.factor
                        ()
ps_samp 
                        <- sample_data
                        (ps) 
                        %>%
  
                        unclass
                        () 
                        %>%
  
                        data.frame
                        () 
                        %>%
  
                        left_join
                        (ps_alpha_div, 
                        by 
                        = 
                        "SampleID"
                        ) 
                        %>%
  
                        melt
                        (
                        measure.vars 
                        = 
                        "Shannon"
                        ,
        
                        variable.name 
                        = 
                        "diversity_measure"
                        ,
        
                        value.name 
                        = 
                        "alpha_diversity"
                        )


                        # reorder's facet from lowest to highest diversity

                        diversity_means 
                        <- 
                        ps_samp 
                        %>%
  
                        group_by
                        (host_subject_id) 
                        %>%
  
                        summarise
                        (
                        mean_div 
                        = 
                        mean
                        (alpha_diversity)) 
                        %>%
  
                        arrange
                        (mean_div)
ps_samp
                        $
                        host_subject_id 
                        <- factor
                        (ps_samp
                        $
                        host_subject_id,
				  diversity_means
                        $
                        host_subject_id)
                    


We use the *nlme* to estimate coefficients for this mixed-effects model.



                        alpha_div_model 
                        <- lme
                        (
                        fixed 
                        = alpha_diversity 
                        ~ 
                        age_binned, 
                        data 
                        = ps_samp,
			  
                        random 
                        = 
                        ~ 
                        1 
                        | 
                        host_subject_id)
                    


**Figure 26.**
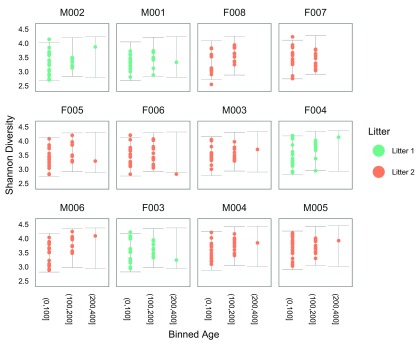
Each point represents the Shannon diversity at one timepoint for a mouse; each panel is a different mouse. The timepoints have been split into three bins, according to the mices’ age. The prediction intervals obtained from mixed-effects modeling are overlaid.

To interpret the results, we compute the prediction intervals for each mouse by age bin combination. These are displayed in Figure 26. The intervals reflect the slight shift in average diversity across ages, but the wide intervals emphasize that more samples would be needed before this observation can be confirmed.



                        new_data 
                        <- expand.grid
                        (
                        host_subject_id 
                        = 
                        levels
                        (ps_samp
                        $
                        host_subject_id),
			   
                        age_binned 
                        = 
                        levels
                        (ps_samp
                        $
                        age_binned))
new_data$
                        pred 
                        <- predict
                        (alpha_div_model, 
                        newdata 
                        = new_data)
X 
                        <- model.matrix
                        (
                        eval
                        (
                        eval
                        (alpha_div_model
                        $
                        call
                        $
                        fixed)[-
                        2
                        ]),
		  new_data[-
                        ncol
                        (new_data)])
pred_var_fixed 
                        <- diag
                        (X 
                        %*% 
                        alpha_div_model
                        $
                        varFix 
                        %*% 
                        t
                        (X))
new_data
                        $
                        pred_var 
                        <- 
                        pred_var_fixed 
                        + 
                        alpha_div_model
                        $
                        sigma 
                        ^ 
                        2
                    




                        # fitted values, with error bars

                        ggplot
                        (ps_samp 
                        %>% 
                        left_join
                        (new_data)) 
                        +
  
                        geom_errorbar
                        (
                        aes
                        (
                        x 
                        = age_binned, 
                        ymin 
                        = pred 
                        - 
                        2 
                        * 
                        sqrt
                        (pred_var),
		      
                        ymax 
                        = pred 
                        + 
                        2 
                        * 
                        sqrt
                        (pred_var)),
		  
                        col 
                        = 
                        "#858585"
                        , 
                        size 
                        = 
                        .1
                        ) 
                        +
  
                        geom_point
                        (
                        aes
                        (
                        x 
                        = age_binned, 
                        y 
                        = alpha_diversity,
		   
                        col 
                        = family_relationship), 
                        size 
                        = 
                        0.8
                        ) 
                        +
  
                        facet_wrap
                        (
                        ~
                        host_subject_id) 
                        +
  
                        scale_y_continuous
                        (
                        limits 
                        = 
                        c
                        (
                        2.4
                        , 
                        4.6
                        ), 
                        breaks 
                        = 
                        seq
                        (
                        0
                        , 
                        5
                        , 
                        .5
                        )) 
                        +
  
                        scale_color_brewer
                        (
                        palette 
                        = 
                        "Set2"
                        ) 
                        +
  
                        labs
                        (
                        x 
                        = 
                        "Binned Age"
                        , 
                        y 
                        = 
                        "Shannon Diversity"
                        , 
                        color 
                        = 
                        "Litter"
                        ) 
                        +
  
                        guides
                        (
                        col 
                        = 
                        guide_legend
                        (
                        override.aes 
                        = 
                        list
                        (
                        size 
                        = 
                        4
                        ))) 
                        +
  
                        theme
                        (
                        panel.border 
                        = 
                        element_rect
                        (
                        color 
                        = 
                        "#787878"
                        , 
                        fill 
                        = 
                        alpha
                        (
                        "white"
                        , 
                        0
                        )),
	 
                        axis.text.x 
                        = 
                        element_text
                        (
                        angle 
                        = 
                        -
                        90
                        , 
                        size 
                        = 
                        6
                        ),
	 
                        axis.text.y 
                        = 
                        element_text
                        (
                        size 
                        = 
                        6
                        ))
                    


### Hierarchical multiple testing

Hypothesis testing can be used to identify individual microbes whose abundance relates to sample variables of interest. A standard approach is to compute a test statistic for each bacteria individually, measuring its association with sample characteristics, and then jointly adjust *p*-values to ensure a False Discovery Rate upper bound. This can be accomplished through the Benjamini-Hochberg procedure, for example ^28^. However, this procedure does not exploit any structure among the tested hypotheses – for example, it is likely that if one Ruminococcus species is strongly associated with age, then others are as well. To integrate this information ^29, 30^, proposed a hierarchical testing procedure, where taxonomic groups are only tested if higher levels are found to be be associated. In the case where many related species have a slight signal, this pooling of information can increase power.

We apply this method to test the association between microbial abundance and age. This provides a complementary view of the earlier analyses, identifying individual bacteria that are responsible for the differences between young and old mice.

**Figure 27.**
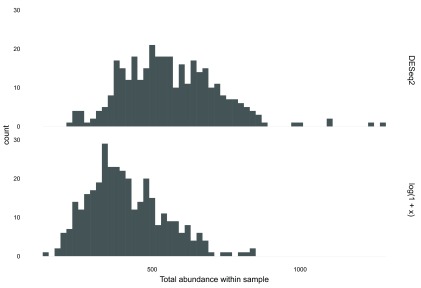
The histogram on the top gives the total DESeq2 transformed abundance within each sample. The bottom histogram is the same as that in Figure 7, and is included to facilitate comparison.

We digress briefly from hierarchical testing to describe an alternative form of count normalization. Rather than working with the logged data as in our earlier analysis, we consider a variance stabilizing transformation introduced by 31 for RNA-seq data and in 32 for 16S rRNA generated count data and available in the *DESeq2* package. The two transformations yield similar sets of significant microbes. One difference is that, after accounting for size factors, the histogram of row sums for DESeq is more spread out in the lower values, refer to Figure 27. This is the motivation of using such a transformation, although for high abundance counts, it is equivalent to the log, for lower and mid range abundances it does not crush the data and yields more powerful results. The code below illustrates the mechanics of computing *DESeq2*’s variance stabilizing transformation on a *phyloseq* object.



                        setup_example
                        (
                        c
                        (
                        "phyloseq"
                        , 
                        "structSSI"
                        , 
                        "plyr"
                        , 
                        "dplyr"
                        , 
                        "reshape2"
                        ,
		  
                        "ggplot2"
                        , 
                        "DESeq2"
                        ))
ps_dds 
                        <- phyloseq_to_deseq2
                        (ps, 
                        ~ 
                        age_binned 
                        + 
                        family_relationship)

                        varianceStabilizingTransformation
                        (ps_dds, 
                        blind 
                        = 
                        TRUE
                        , 
                        fitType 
                        = 
                        "parametric"
                        )
                    




                        ## class: DESeqTransform
## dim: 389 344
## metadata(1): version
## assays(1): ''
## rownames(389):
##   GCGAGCGTTATCCGGATTTATTGGGTTTAAAGGGTGCGCAGGCGGAAGATCAAGTCAGCGGTAAAATTGAGAGGCTCAACCTCTTCGAGCCGTTGAAACTGGTTTTC
##   GCGAGCGTTATCCGGATTTATTGGGTTTAAAGGGTGCGCAGGCGGACTCTCAAGTCAGCGGTCAAATCGCGGGGCTCAACCCCGTTCCGCCGTTGAAACTGGGAGCC
##   ...
##   GCTAGCGTTGTTCGGAATTACTGGGCGTAAAGCGCGTGTAGGCGGTTTGCCAAGTTGGGTGTGAAAGCCTTGAGCTCAACTCAAGAAATGCACTCAGTACTGG
##   GCAAGCGTTACTCGGAATCACTGGGCGTAAAGAGCGCGTAGGCGGGATAGTCAGTCAGGTGTGAAATCCTATGGCTTAACCATAGAACTGCATTTGAAACTAC
## rowData names(5): baseMean baseVar allZero dispGeneEst dispFit
## colnames(344): F3D0 F3D1 ... M6D8 M6D9
## colData names(17): collection_date biome ... age_binned sizeFactor
                    




                        ps_dds 
                        <- estimateSizeFactors
                        (ps_dds)
ps_dds 
                        <- estimateDispersions
                        (ps_dds)
abund 
                        <- getVarianceStabilizedData
                        (ps_dds)
                    


We use the *structSSI* to perform the hierarchical testing ^33^. For more convenient printing, we first shorten the names of each microbe.



                        short_names 
                        <- substr
                        (
                        rownames
                        (abund), 
                        1
                        , 
                        5
                        )
                        %>%
  
                        make.names
                        (
                        unique 
                        = 
                        TRUE
                        )

                        rownames
                        (abund) 
                        <- 
                        short_names
                    


Unlike standard multiple hypothesis testing, the hierarchical testing procedure needs univariate tests for each higher-level taxonomic group, not just every bacteria. A helper function, treePValues, is available for this; it expects an edgelist encoding parent-child relationships, with the first row specifying the root node.



                        el 
                        <- phy_tree
                        (pslog)
                        $
                        edge
el0 
                        <- 
                        el
el0 
                        <- 
                        el0[
                        nrow
                        (el)
                        :
                        1
                        , ]
el_names 
                        <- c
                        (short_names, 
                        seq_len
                        (
                        phy_tree
                        (pslog)
                        $
                        Nnode))
el[, 
                        1
                        ] 
                        <- 
                        el_names[el0[, 
                        1
                        ]]
el[, 
                        2
                        ] 
                        <- 
                        el_names[
                        as.numeric
                        (el0[, 
                        2
                        ])]
unadj_p 
                        <- treePValues
                        (el, abund, 
                        sample_data
                        (pslog)
                        $
                        age_binned)
                    


We can now correct *p*-value using the hierarchical testing procedure. The test results are guaranteed to control several variants of FDR control, but at different levels; we defer details to 29, 30, 33.



                        hfdr_res 
                        <- hFDR.adjust
                        (unadj_p, el, 
                        .75
                        )

                        summary
                        (hfdr_res)
                    




                        ## Number of hypotheses: 776
## Number of tree discoveries: 461
## Estimated tree FDR: 1
## Number of tip discoveries: 219
## Estimated tips FDR: 1
##
##  hFDR adjusted p-values:
##             unadjp       adjp adj.significance
## GCAAG.71  1.01e-67   2.02e-67              ***
## GCAAG.96  1.33e-67   2.65e-67              ***
## GCAAG.190 1.10e-58   2.21e-58              ***
## GCAAG.254 2.01e-48   4.03e-48              ***
## GCAAG.150 4.90e-46   9.80e-46              ***
## GCGAG.2   5.28e-38   1.06e-37              ***
## GCAAG.170 6.54e-38   1.31e-37              ***
## GCAAG.1   1.16e-35   2.32e-35              ***
## GCAAG.146 4.83e-33   9.66e-33              ***
## GCGAG.21  1.40e-28   2.79e-28              ***
                    




                        abund_sums 
                        <- rbind
                        (
                        data.frame
                        (
                        sum 
                        = 
                        colSums
                        (abund),
			          
                        sample 
                        = 
                        colnames
                        (abund),
			          
                        type 
                        = 
                        "DESeq2"
                        ),
		      
                        data.frame
                        (
                        sum 
                        = 
                        rowSums
                        (
                        otu_table
                        (pslog)),
			          
                        sample 
                        = 
                        rownames
                        (
                        otu_table
                        (pslog)),
			          
                        type 
                        = 
                        "log(1 + x)"
                        ))

                        ggplot
                        (abund_sums) 
                        +
  
                        geom_histogram
                        (
                        aes
                        (
                        x 
                        = sum), 
                        binwidth 
                        = 
                        20
                        ) 
                        +
  
                        facet_grid
                        (type 
                        ~ 
                        .) 
                        +
  
                        xlab
                        (
                        "Total abundance within sample"
                        )
                    


**Figure 28.**
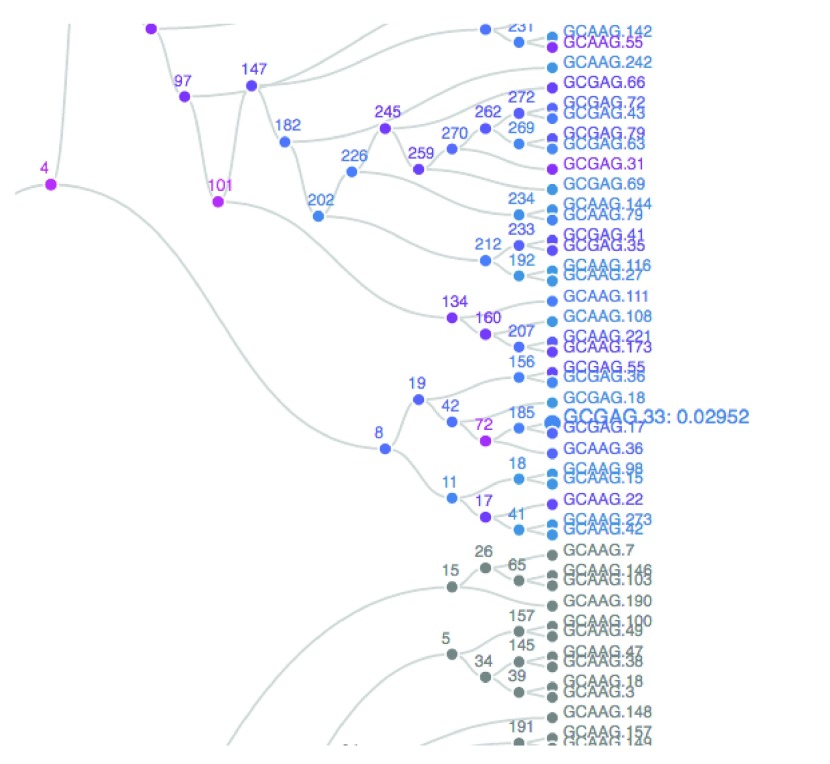
A screenshot of a subtree with many differentially abundant bacteria, as determined by the hierarchical testing procedure. Currently the user is hovering over the node associated with bacteria GCGAG.33; this causes the adjusted *p*-value (0.0295) to appear.



                        ## [only 10 most significant hypotheses shown]
## ---
## Signif. codes: 0 '***' 0.015 '**' 0.15 '*' 0.75 '.' 1.5 '-' 1
                    




                        plot
                        (hfdr_res, 
                        height 
                        = 
                        5000
                        ) 
                        # opens in a browser
                    


The plot opens in a new browser – a static screenshot of a subtree is displayed in Figure 28. Nodes are shaded according to *p*-values, from blue to orange, representing the strongest to weakest associations. Grey nodes were never tested, to focus power on more promising subtrees. Scanning the full tree, it becomes clear that the association between age group and bacterial abundance is present in only a few isolated taxonomic groups, but that it is quite strong in those groups. To give context to these results, we can retrieve the taxonomic identity of the rejected hypotheses.



                        options
                        (
                        width
                        =
                        100
                        )

                        tax 
                        <- tax_table
                        (pslog)[, 
                        c
                        (
                        "Family"
                        , 
                        "Genus"
                        )] 
                        %>%
  
                        data.frame
                        ()
tax
                        $
                        seq 
                        <- 
                        short_names
                    




                        hfdr_res
                        @
                        p.vals
                        $
                        seq 
                        <- rownames
                        (hfdr_res
                        @
                        p.vals
                        )
tax 
                        %>%
  
                        left_join
                        (hfdr_res
                        @
                        p.vals
                        ) 
                        %>%
  
                        arrange
                        (adjp) 
                        %>% 
                        head
                        (
                        10
                        )
  

                        ## 	          Family            Genus        seq     unadjp      adjp  adj.significance
## 1     Lachnospiraceae        Roseburia   GCAAG.71   1.01e-67  2.02e-67               ***
## 2     Lachnospiraceae             <NA>   GCAAG.96   1.33e-67  2.65e-67               ***
## 3     Lachnospiraceae Clostridium_XlVa  GCAAG.190   1.10e-58  2.21e-58               ***
## 4     Lachnospiraceae             <NA>  GCAAG.254   2.01e-48  4.03e-48               ***
## 5     Lachnospiraceae Clostridium_XlVa  GCAAG.150   4.90e-46  9.80e-46               ***
## 6  Porphyromonadaceae             <NA>    GCGAG.2   5.28e-38  1.06e-37               ***
## 7     Lachnospiraceae Clostridium_XlVa  GCAAG.170   6.54e-38  1.31e-37               ***
## 8                <NA>             <NA>    GCAAG.1   1.16e-35  2.32e-35               ***
## 9     Lachnospiraceae             <NA>  GCAAG.146   4.83e-33  9.66e-33               ***
## 10 Porphyromonadaceae             <NA>   GCGAG.21   1.40e-28  2.79e-28               ***
                    


It seems that the most strongly associated bacteria all belong to family *Lachnospiraceae*, which is consistent with the random forest results in Section.

### Multitable techniques

Many microbiome studies attempt to quantify variation in the microbial, genomic, and metabolic measurements across different experimental conditions. As a result, it is common to perform multiple assays on the same biological samples and ask what features – bacteria, genes, or metabolites, for example – are associated with different sample conditions. There are many ways to approach these questions, which to apply depends on the study’s focus.

Here, we will focus on one specific workflow that uses sparse Canonical Correlation Analysis (sparse CCA), a method well-suited to both exploratory comparisons between samples and the identification of features with interesting variation. We will use an implementation from the *PMA* ^34^.

Since the mouse data used above included only a single table, we use a new data set, collected by the study ^35^. There are two tables here, one for bacteria and another with metabolites. 12 samples were obtained, each with measurements at 637 m/z values and 20,609 OTUs; however, about 96% of the entries of the microbial abundance table are exactly zero. The code below retrieves this data.



                        setup_example
                        (
                        c
                        (
                        "phyloseq"
                        , 
                        "ggplot2"
                        , 
                        "reshape2"
                        , 
                        "ade4"
                        , 
                        "PMA"
                        ,
		  
                        "genefilter"
                        , 
                        "ggrepel"
                        ))
                    




                        metab_path 
                        <- 
                        "data/metabolites.csv"

                        microbe_path 
                        <- 
                        "data/microbe.rda"

                        metab 
                        <- read.csv
                        (metab_path, 
                        row.names 
                        = 
                        1
                        )

                        metab 
                        <- as.matrix
                        (metab)

                        microbe 
                        <- get
                        (
                        load
                        (microbe_path))
                    


Our preprocessing mirrors that done for the mouse data. We first filter down to microbes and metabolites of interest, removing those that are zero across many samples. Then, we transform them to weaken the heavy tails.



                        keep_ix 
                        <- rowSums
                        (metab 
                        == 
                        0
                        ) 
                        <= 
                        3

                        metab 
                        <- 
                        metab[keep_ix, ]

                        microbe 
                        <- prune_taxa
                        (
                        taxa_sums
                        (microbe) 
                        > 
                        4
                        , microbe)

                        microbe 
                        <- filter_taxa
                        (microbe, 
                        filterfun
                        (
                        kOverA
                        (
                        3
                        , 
                        2
                        )), 
                        TRUE
                        )
                    




                        metab 
                        <- log
                        (
                        1 
                        + 
                        metab, 
                        base 
                        = 
                        10
                        )

                        X 
                        <- otu_table
                        (microbe)
                        @
                        .Data

                        X[X 
                        > 
                        50
                        ] 
                        <- 
                        50

                        
                    


We can now apply sparse CCA. This method compares sets of features across high-dimensional data tables, where there may be more measured features than samples. In the process, it chooses a subset of available features that capture the most covariance – these are the features that reflect signals present across multiple tables. We then apply PCA to this selected subset of features. In this sense, we use sparse CCA as a screening procedure, rather than as an ordination method.

Our implementation is below. The parameters penaltyx and penaltyz are sparsity penalties. Larger values of penaltyx will result in fewer selected microbes, similarly penaltyz modulates the number of selected metabolites. We tune them manually to facilitate subsequent interpretation – we generally prefer more sparsity than the default parameters would provide.



                        cca_res 
                        <- CCA
                        (
                        t
                        (X), 
                        t
                        (metab),  
                        penaltyx 
                        = 
                        .15
                        , 
                        penaltyz 
                        = 
                        .15
                        )

## 123456789101112131415

cca_res

## Call: CCA(x = t(X), z = t(metab), penaltyx = 0.15, penaltyz = 0.15)
##
##
## Num non-zeros u's: 5
## Num non-zeros v's: 15
## Type of x: standard
## Type of z: standard
## Penalty for x: L1 bound is 0.15
## Penalty for z: L1 bound is 0.15
## Cor(Xu,Zv): 0.974
                    


With these parameters, 5 microbes and 15 metabolites have been selected, based on their ability to explain covariation between tables. Further, these 20 features result in a correlation of 0.974 between the two tables. We interpret this to mean that the microbial and metabolomic data reflect similar underlying signals, and that these signals can be approximated well by the 20 selected features. Be wary of the correlation value, however, since the scores are far from the usual bivariate normal cloud. Further, note that it is possible that other subsets of features could explain the data just as well – sparse CCA has minimized redundancy across features, but makes no guarantee that these are the “true” features in any sense.

Nonetheless, we can still use these 20 features to compress information from the two tables without much loss. To relate the recovered metabolites and OTUs to characteristics of the samples on which they were measured, we use them as input to an ordinary PCA.



                        combined 
                        <- cbind
                        (
                        t
                        (X[cca_res
                        $
                        u 
                        !
                        = 
                        0
                        , ])
                        ,
		    
                        t
                        (
                        metab[cca_res
                        $
                        v 
                        !
                        = 
                        0
                        , ]))

                        pca_res 
                        <- dudi.pca
                        (combined, 
                        scannf 
                        = F, 
                        nf 
                        = 
                        3
                        )
                    




                        # annotation

                        genotype 
                        <- substr
                        (
                        rownames
                        (pca_res
                        $
                        li), 
                        1
                        , 
                        2
                        )

                        sample_type 
                        <- substr
                        (
                        rownames
                        (pca_res
                        $
                        l1
                        ), 
                        3
                        , 
                        4
                        )

                        feature_type 
                        <- grepl
                        (
                        "\\."
                        , 
                        colnames
                        (combined))

                        feature_type 
                        <- ifelse
                        (feature_type, 
                        "Metabolite"
                        , 
                        "OTU"
                        )


                        sample_info 
                        <- data.frame
                        (pca_res
                        $
                        li, genotype, sample_type)

                        feature_info 
                        <- data.frame
                        (pca_res
                        $
                        c1,
			      
                        feature 
                        = 
                        substr
                        (
                        colnames
                        (combined), 
                        1
                        , 
                        6
                        ))
                    


**Figure 29.**
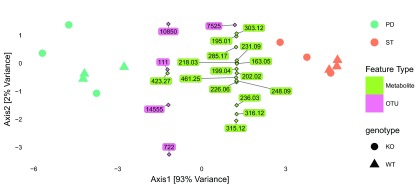
A PCA triplot produced from the CCA selected features in from muliple data types (metabolites and OTUs). Note that we have departed from our convention of fixing the aspect ratio here as the second axis represents very little of the variability and the plot would actually become unreadable.

Figure 29 displays a PCA *triplot*, where we show different types of samples and the multidomain features (Metabolites and OTUs). This allows comparison across the measured samples – triangles for Knockout and circles for wild type – and characterizes the influence the different features – diamonds with text labels. For example, we see that the main variation in the data is across PD and ST samples, which correspond to the different diets. Further, large values of 15 of the features are associated with ST status, while small values for 5 of them indicate PD status. The advantage of the sparse CCA screening is now clear – we can display most of the variation across samples using a relatively simple plot, and can avoid plotting the hundreds of additional points that would be needed to display all of the features.



                        ggplot
                        () 
                        + 
                        geom_point
                        (
                        data 
                        = sample_info,
            
                        aes
                        (
                        x 
                        = Axis1, 
                        y 
                        = Axis2, 
                        col 
                        = sample_type, 
                        shape 
                        = genotype), 
                        size 
                        = 
                        3
                        ) 
                        +
  
                        geom_label_repel
                        (
                        data 
                        = feature_info,
                     
                        aes
                        (
                        x 
                        = 
                        5.5 
                        * 
                        CS1, 
                        y 
                        = 
                        5.5 
                        * 
                        CS2, 
                        label 
                        = feature, 
                        fill 
                        = feature_type),
                     
                        size 
                        = 
                        2
                        , 
                        segment.size 
                        = 
                        0.3
                        ,
                     
                        label.padding 
                        = 
                        unit
                        (
                        0.1
                        , 
                        "lines"
                        ), 
                        label.size 
                        = 
                        0
                        ) 
                        +
  
                        geom_point
                        (
                        data 
                        = feature_info,
	      
                        aes
                        (
                        x 
                        = 
                        5.5 
                        * 
                        CS1, 
                        y 
                        = 
                        5.5 
                        * 
                        CS2, 
                        fill 
                        = feature_type),
	      
                        size 
                        = 
                        1
                        , 
                        shape 
                        = 
                        23
                        , 
                        col 
                        = 
                        "#383838"
                        ) 
                        +
  
                        scale_color_brewer
                        (
                        palette 
                        = 
                        "Set2"
                        ) 
                        +
  
                        scale_fill_manual
                        (
                        values 
                        = 
                        c
                        (
                        "#a6d854"
                        , 
                        "#e78ac3"
                        )) 
                        +
  
                        guides
                        (
                        fill 
                        = 
                        guide_legend
                        (
                        override.aes 
                        = 
                        list
                        (
                        shape 
                        = 
                        32
                        , 
                        size 
                        = 
                        0
                        ))) 
                        +
  
                        coord_fixed
                        (
                        sqrt
                        (pca_res
                        $
                        eig[
                        2
                        ] 
                        / 
                        pca_res
                        $
                        eig[
                        2
                        ])) 
                        +
  
                        labs
                        (
                        x 
                        = 
                        sprintf
                        (
                        "Axis1 [%s%% Variance]"
                        ,
	            
                        100 
                        * 
                        round
                        (pca_res
                        $
                        eig[
                        1
                        ] 
                        / 
                        sum
                        (pca_res
                        $
                        eig), 
                        2
                        )),
       
                        y 
                        = 
                        sprintf
                        (
                        "Axis2 [%s%% Variance]"
                        ,
	            
                        100 
                        * 
                        round
                        (pca_res
                        $
                        eig[
                        2
                        ] 
                        / 
                        sum
                        (pca_res
                        $
                        eig), 
                        2
                        )),
       
                        fill 
                        = 
                        "Feature Type"
                        , 
                        col 
                        = 
                        "Sample Type"
                        )
                    


### Operation

The programs and source for this article can be run using version 3.3 of R and version 3.3 of Bioconductor.

## Conclusions

We have shown how a complete workflow in R is now available to denoise, identify and normalize next generation amplicon sequencing reads using probabilistic models with parameters fit using the data at hand.

We have provided a brief overview of all the analyses that become possible once the data has been imported into the R environment. Multivariate projections using the phylogenetic tree as the relevant distance between OTUs/RSVs can be done using weighted unifrac or double principal coordinate analyses using the *phyloseq* package. Biplots provide the user with an interpretation key. These biplots have been extended to triplots in the case of multidomain data incorporating genetic, metabolic and taxa abundances. We illustrate the use of network based analyses, whether the community graph is provided from other sources or from a taxa co-occurrence computation using a Jaccard distance.

We have briefly covered a small example of using three supervised learning functions (random forests, partial least squares and) to predict a response variable,

The main challenges in tackling microbiome data come from the many different levels of heterogeneity both at the input and output levels. These are easily accommodated through R’s capacity to combine data into S4 classes. We are able to include layers of information, trees, sample data description matrices, contingency table in the phyloseq data sctructures. The plotting facilities of *ggplot2* and *ggnetwork* allow for the layering of information in the output into plots that combine graphs, multivariate information and maps of the relationships between covariates and taxa abundances. The layering concept allows the user to provide reproducible publication level figures with multiple heterogeneous sources of information. Our main goal in providing these tools has been to enhance the statistical power of the analyses by enabling the user to combine frequencies, quality scores and covariate information into complete and testable projections.

## Summary

This illustration of possible workflows for microbiome data combining trees, networks, normalized read counts and sample information showcases the capabilities and reproducibility of an R based system for analysing bacterial communities. We have implemented key components in C wrapped within the Bioconductor package *dada2* to enable the different steps to be undertaken on a laptop.

Once the sequences have been filtered and tagged they can be assembled into a phylogenetic tree directly in R using the maximum likelihood tree estimation available in *phangorn*. The sequences are then assembled into a phyloseq object containing all the sample covariates, the phylogenetic tree and the sample-taxa contingency table.

These data can then be visualized interactively with Shiny-phyloseq, plotted with one line wrappers in phyloseq and filtered or transformed very easily.

The last part of the paper shows more complex analyses that require direct plotting and advanced statistical analyses.

Multivariate ordination methods allow useful lower dimensional projections in the presence of phylogenetic information or multidomain data as shown in an example combining metabolites, OTU abundances,

Supervised learning methods provide lists of the most relevant taxa in discriminating between groups.

Bacterial communities can be represented as co-occurrence graphs using network based plotting procedures available in R. We have also provided examples where these graphs can be used to test community structure through non parametric permutation resampling. This provides implementations of the Friedman Rafsky ^25^ tests for microbiome data which have not been published previously.

## Data availability

The data referenced by this article are under copyright with the following copyright statement: Copyright: © 2016 Callahan BJ et al.

Intermediary data for the analyses are made available both on GitHub at https://github.com/spholmes/F1000_workflow and at the Stanford digital repository permanent url for this paper: http://purl.stanford.edu/wh250nn9648. All other data have been previously published and the links are included in the paper.

## Software availability

Bioconductor packages at https://www.bioconductor.org/. CRAN packages at https://cran.r-project.org/.

Permanent repository for the data and program source of this paper: https://purl.stanford.edu/wh250nn9648

Latest source code as at the time of publication: https://github.com/spholmes/F1000_workflow

Archived source as at the time of publication: Zenodo: F1000_workflow: MicrobiomeWorkflowv0.9, doi: 10.5281/zenodo.54544 ^36^
