# Supplementary material for: Bioconductor Workflow for Microbiome Data Analysis: from raw reads to community analyses
Source: F1000Res. 2016 Nov 2;5:1492. Originally published 2016 Jun 24. [Version 2] doi: 10.12688/f1000research.8986.2 (PMC4955027; doi:10.12688/f1000research.8986.2)
Supplement: Peer review history

# Peer review: Referee response for version 2

**DOI:** 10.5256/f1000research.10545.r17380

**Published:** 2016-11-07

**Authors:** Leo Lahti

**Affiliations:** 1 Department of Mathematics and Statistics, University of Turku, Turku, Finland

The title and abstract appropriately summarize the contents, and the text is fluent to read. The manuscript aims to cover a vast area, and does good job in summarizing the relevant key aspects in a single workflow.

 The study design, methods and analysis and their suitability are properly described with appropriate references. The work describes a recommendation for a workflow based on the author's comprehensive experience in this field. It does not provide thorough comparison or benchmarking of the methods but relevant research is cited and key comparisons are already available in the literature. The contribution in this work is to combine the individual elements into a coherent workflow that describes very typical steps and recommended choices in standard taxonomic analysis.

 * Major comments:

 The main shortcoming is that the workflow is not provided in a readily reproducible format. I cloned the github repository, and could run the analysis rnw files (PartI, PartII, PartIII) individually, as well as the main.rnw. These result in .text file but using pdflatex or latex could not be readily applied to convert these into the final PDF format. The github site does not mention how the rnw files should actually be converted into PDF. This is not evident as there are many ways to do this and the success will depend on the overall setup. The authors could include for instance a simple shell script or README in the github main directory, showing what steps are taken to get from the original R/Rnw files to the final PDF reports. This would greatly increase the utility and reproducibility of this work.

 * Minor comment:

 - Something is missing from Conclusions. There is paragraph that in its entirety reads as follows: "We have briefly covered a small example of using three supervised learning functions (random forests, partial least squares and) to predict a response variable," - it misses text in parentheses and ends with a comma.

 - Could you cite or discuss in more detail based on your experience whether Friedman Rafsky method outperforms alternative or at least closely related methods in the pairwise comparison task ? Not required but would be interesting to know.

 - The phyloseq package might serve its purpose better if split in smaller and more compact packages. The class structure is really useful and valuable, and would deserve its own package. This would better serve the overall microbiome data analytics community which can build on this and expand phyloseq capabilities in separate packages, in the same way as certain microarray data structures became a norm with the RMA and limma packages, with subsequent explosion in analysis methodologies. I am here just repeating my comment from the first review. Not required for this manuscript, however.

I have read this submission. I believe that I have an appropriate level of expertise to confirm that it is of an acceptable scientific standard.

# Peer review: Referee response for version 1

**DOI:** 10.5256/f1000research.9666.r14571

**Published:** 2016-08-08

**Authors:** Nandita R. Garud

**Affiliations:** 1 Gladstone Institutes, University of California, San Francisco, San Francisco, CA, USA

This article is a valuable resource for the metagenomics field. The thorough examples of several statistical analyses of metagenomic data will help both the novice and expert in analyzing their own data. Additionally, this paper sets a standard in the field for documenting analyses.

 Both DADA2 and PhyloSeq have much to offer. DADA2 identifies OTUs, which are termed in this paper ‘Ribosomal Sequence Variants,’ reflecting the extra granularity with which DADA2 is capable of resolving OTUs. The RSVs identified by DADA2 offer the ability to conduct higher resolution analyses on 16S data. PhyloSeq is comprised of numerous capabilities to analyze metagenomic data, making it quite easy for a user to load and analyze their data.

 Below I make a few suggestions for clarification purposes. I enjoyed reading this article and have already benefited greatly from using DADA2 and PhyloSeq in my own work.

 Minor critiques and suggestions:

- A very attractive feature of DADA2 is its ability to resolve RSVs. I wonder if the authors could expand more on the findings they have made with the higher resolution OTUs found by DADA2. This would highlight why DADA2 is such a powerful tool.
- I wonder if the examples that the authors provide could be more biologically motivated. For example, could the authors explain the mouse data set in greater depth in the introduction? What did Kozich *et al. *2013 and Schloss *et al. *2012 find in these data sets? Were DADA2 and PhyloSeq used to analyze the data in these two papers? If not, are the findings different? I enjoyed reading about the different metagenomic properties of mice of different ages. More description along these lines in the introduction would make it motivating to understand why the various preprocessing steps are done and an overview of what is to come.
- Page 4 – it could be helpful to illustrate some of the properties of the software with numbers and data. For example, DADA2 has the ability to infer OTUs from pooled or unpooled data. Could the authors illustrate the number of RSVs found in the two scenarios?
- Figure 2 -- Could the authors explain on Page 4 what sequencing error rates are being inferred (i.e. transition and transversion errors)? Which parameters are inferred to come up with the solid black line? An explicit reference to Figure 2 in the text could help. Additionally, headers indicating Forward and Reverse reads in Figure 2 could help to distinguish the plots.
- Page 6 – Is the multiple sequence alignment feature capable of multiple methods? If so, do you advocate for using ClustalW for metageonomic data? Why?
- Page 6 -- Could the authors define what a GTR+G+I model is?
- I wonder if the authors could give some more guidance on how to construct the PhyloSeq object from scratch without relying import functions. For example, I tried making a PhyloSeq object using Metaphlan2 output. Unfortunately I could not figure out how to merge Metaphlan2 biom files for each sample, and so I had to fiddle with Phyloseq for sometime to manually create the OTU, sample, and taxa tables for multiple samples.

I have read this submission. I believe that I have an appropriate level of expertise to confirm that it is of an acceptable scientific standard.

# Response

**Published:** 2016-11-04

**Authors:** Benjamin Callahan

**Affiliations:** Stanford University, USA

Thanks for your comments and suggestions. We made several improvements to the revised manuscript in response:

 We added an explicit reference to Figure 2 in the text. The error rates being estimated in each plot are indicated in the text just above each plot. A2C (A to C) is shorthand for an A being converted to a C by errors in the amplicon sequencing process.

 We changed the multiple-sequence alignment method in the workflow to that implemented by the DECIPHER package, largely because of its improved computational performance.

 We added a brief text description of GTR+G+I (Generalized time-reversible with Gamma rate variation).

 We did not expand our evaluation of RSVs vs. OTUs or pooled vs. unpooled inference. Performing such evaluations well is a significant undertaking and would take significant space to explain, and our primary purpose here is to demonstrate the many features of an R/Bioconductor amplicon analysis workflow.

 For evaluation of DADA2, our manuscript introducing the method examines differences between the output of DADA2 and OTU methods and we are writing another manuscript that looks at performance on datasets with many samples. On the issue of pooled vs. unpooled results, the short answer is that we find both approaches work well. If just counting the number of output OTU sequences, pooled inference generally finds more because of its higher sensitivity to sequences that are found in many samples but are rare in each. Of note, we generally find these pooled-only sequences to be very highly enriched for contaminants (eg. kit contaminants), which are expected to distributed in just this way.

 We also did not expand much on the biological findings from this dataset in the initial paper (Stabilization of the murine gut microbiome following weaning, Schloss et al. 2012), as they were quite limited, essentially boiling down to the observation that gut sample early in life differed by more on average than samples from later in life. However, the dataset has been used in a number of studies as an example dataset for testing new methods (as in Kozich et al. 2013) and that is the way in which we are using it here.

# Peer review: Referee response for version 1

**DOI:** 10.5256/f1000research.9666.r14570

**Published:** 2016-07-18

**Authors:** Zachary Charlop-Powers

**Affiliations:** 1 Laboratory of Genetically Encoded Small Molecules, The Rockefeller University, New York, NY, USA

There is a growing push in the computational sciences for adopting software practices that promote replicability and provide methodological transparency. In the field of microbiome research these practices should minimize the standard culprits of error-creep such as file proliferation, and incompatible formats; they should provide sound default choices for the core computational steps of sequence clustering and taxonomic assignment; and they should facilitate reproducible statistical analyses of the resulting data. By providing a step-by-step analysis of a microbiome dataset that can be completed entirely from within the R statistical computing environment, this workflow does an admirable job of bringing these best-practices to the world of microbiome science.

 The article takes a reader through the steps of processing raw sequence data and loading the data into R. It then demonstrates how to use basic exploratory data analysis to get a sense of the data and finally introduces the use of various statistical packages to search-for and validate patterns. The majority of the article focuses on the application of statistical concepts  to microbiome data and this is where scientists would like to be spending their time. However, this allocation of ink-space is only possible because the recent release of the DADA2 package allows the authors (and subsequent users) to condense all the read-processing portion of the tutorial into a few short steps.  DADA2 provides a new and arguably superior method for clustering raw amplicon reads and, by processing the reads and assigning taxonomy, it fills in the computational gap required to work completely within R.  The benefits of this workflow are fairly self-evident in the amount of space in their workflow devoted to data processing versus exploration, however, there are other benefits as well, of which I will name two. First, by using packages hosted on CRAN or Bioconductor, the authors can leverage the Bioconductor build system and ensure a fully working environment, a non-trivial prerequisite in a field with myriad tools. Second, by providing an integrated set of tools there are few, if any, intermediate files required to analyze a dataset. In addition to reducing the cognitive burden of a newcomer, this generally reduces the footprint for errors.

 This article is an excellent introduction on how to process and analyze a 16S amplicon dataset. Because of the relative ease of working entirely within a single environment, and for the sound design principles used by the core R packages in this analysis, I predict this workflow will become a useful resource, if not a direct template, for many microbiome scientists learning to process their data.

I have read this submission. I believe that I have an appropriate level of expertise to confirm that it is of an acceptable scientific standard.

# Peer review: Referee response for version 1

**DOI:** 10.5256/f1000research.9666.r14572

**Published:** 2016-07-08

**Authors:** Leo Lahti

**Affiliations:** 1 Department of Mathematics and Statistics, University of Turku, Turku, Finland

This work reports a standard R/Bioconductor open source workflow for the analysis of microbial community profiling data based on (Illumina MiSeq) 16S rRNA amplicon sequencing. The main contribution of the

 paper is to present a compact overview of a typical microbiome analysis workflow in R, and to integrate accumulated knowledge by the authors regarding best practices in microbiome bioinformatics based on

 the R statistical programming environment.

 The workflow covers key steps from raw sequencing data prepreprocessing to standard statistical testing, data integration, and visualization. The methodologies are rigorous, and represent a straightforward combination of previously published R tools that are among the state-of-the-art in the field. Reliance on Bioconductor packages provides further guarantees for high quality of the software components. All data and code underlying the paper are openly available, and I was also able replicate the complete workflow after some initial setups. I examined about half of the examples in more detail, and could reproduce manuscript figures in all cases that I tested.

 No new methods are introduced, and the main contribution of the work is to showcase good statistical practice based on existing software components, some of which have been previously published by the authors of this manuscript. Appropriate references are provided throughout the text. Such overview papers are useful, however, as they can provide benchmarks and recommendations on complete workflows,

 where the different analysis steps are not independent in any real study and deserve analysis in their own right.

 The analysis steps are explained in clear language and with sufficient detail. The work is technically sound. The main drawback is that the manuscript is somewhat scattered as it aims to cover a large and versatile set of tools in a single paper. The quality of the analysis is high, and the overview is useful, and the paper could be accepted after taking into account my comments below.

**Major comments**

- The work is somewhat scattered due to the wide coverage. The paper could benefit from having less figures and and increasing focus on key aspects. For instance the number of biplots and network figures could be reduced. The data integration part (CCA etc.) is useful but very brief and probably difficult to comprehend by readers who are new to those approaches. I would recommend either cutting or expanding this part and also otherwise checking if the manuscript can be made more compact by removing some examples (perhaps by moving some examples into supplementary material or online documentation?).
- The examples with DADA2 and the hierarchical testing procedure are particularly useful; these recently published methods would deserve to become more widely used. Sufficient details have been given for this work.
- Instructions on how to exactly use the source files provided in Github are missing. The rnw files are missing latex headers so I could not readily generate final readable reports from the rnw files. The code itself was clear, and after some setups I could replicate all analyses after changing some path definitions and running the code interactively on R command line. But this relied on my earlier good knowledge on R and automated document generationsystems. Users who are less experienced with these tools would benefit from improved instructions on how to run the workflow. The  README.md file in Github should give more detailed instructions (or link to instructions) on how to exactly reproduce the complete example workflow and generate the final reports.
- In the "Infer sequence variants" section it is mentioned that "Sequence inference removed nearly all substitution and indel errors from the data". How this was quantified to reach this conclusion?

**Minor comments**

- The phyloseq R package has been published earlier and represents an extremely useful class structure for microbiome profiling data that has high potential of becoming a popular standard in R. These tools, and their (online) documentation form essential background material for this manuscript. Better separaration of the data structures and tools in this manuscript, the R packages (in particular phyloseq) and their documentation. This would make it easier for the wider R community to build on this work and contribute further tools that take advantage of the phyloseq data structure. This is not required for this manuscript but a suggestion for improvement.
- I had to investigate the code a while to see that the file http://www.mothur.org/MiSeqDevelopmentData/StabilityNoMetaG.tar has to be stored in a data/MiSeq_SOP/ directory after download and extraction. Not a big deal but it would be even more handy to have a download script (R or shell) available in the F1000_workflow/data/ directory, and the instructions would then give clear advice on how to automate the complete analysis workflow. To streamline the workflow example, consider providing some example data sets as R data packages.
- At the github repo README.md the command knit("PartIIphyloseq.Rnw") should be knit("PartIIphyloseq.rnw")
- In PartIIIanalysis.rnw the script gets stuck at:
  options(digits = 3, width = 80,prompt = " ", continue = " ")
  (I was waiting 24 hours; then restarted and tried again with same result).
  Therefore I skipped this row in my tests. Please fix.
- Is it intentional that figures 8, 10, 11, 12, 13, 14 and some other figures have an unbalanced width/height ratio? The figures might seem more clear if the width/height ratio was more balanced.
- The plot_abundance function could be readily provided in the phyloseq package?
- Quality of Figure 1 is relatively poor and could be improved.
- Figure 31: in title: fix "muliple" into "multiple"

I have read this submission. I believe that I have an appropriate level of expertise to confirm that it is of an acceptable scientific standard.
